# Genetic and Non-Genetic Determinants of Raltegravir Penetration into Cerebrospinal Fluid: A Single Arm Pharmacokinetic Study

**DOI:** 10.1371/journal.pone.0082672

**Published:** 2013-12-11

**Authors:** Daniel H. Johnson, Deborah Sutherland, Edward P. Acosta, Husamettin Erdem, Danielle Richardson, David W. Haas

**Affiliations:** 1 Department of Medicine, Vanderbilt University School of Medicine, Nashville, Tennessee, United States of America; 2 Department of Pharmacology, Vanderbilt University School of Medicine, Nashville, Tennessee, United States of America; 3 Department of Pathology, Microbiology & Immunology, Vanderbilt University School of Medicine, Nashville, Tennessee, United States of America; 4 Center for Human Genetics Research, Vanderbilt University School of Medicine, Nashville, Tennessee, United States of America; 5 Department of Medicine, University of Alabama at Birmingham, Birmingham, Alabama, United States of America; Temple University School of Medicine, United States of America

## Abstract

**Background:**

Antiretroviral drugs vary in their central nervous system penetration, with better penetration possibly conferring neurocognitive benefit during human immunodeficiency virus (HIV) therapy. The efflux transporter gene *ABCB1* is expressed in the blood-brain barrier, and an *ABCB1* variant (3435C→T) has been reported to affect *ABCB1* expression. The integrase inhibitor raltegravir is a substrate for ABCB1. We examined whether *ABCB1* 3435C→T affects raltegravir disposition into cerebrospinal fluid (CSF), and explored associations with polymorphisms in other membrane transporter genes expressed in the blood-brain barrier.

**Methods:**

Forty healthy, HIV-negative adults of European descent (20 homozygous for *ABCB1* 3435 C/C, 20 homozygous for 3435 T/T, each group divided equally between males and females) were given raltegravir 400 mg twice daily for 7 days. With the final dose, plasma was collected for pharmacokinetic analysis at 9 timepoints over 12 hours, and CSF collected 4 hours post dose.

**Results:**

The 4-hour CSF concentration correlated more strongly with 2-hour (r^2^=0.76, P=1.12x10^-11^) than 4-hour (r^2^=0.47, P=6.89x10^-6^) single timepoint plasma concentration, and correlated strongly with partial plasma area-under-the-curve values (AUC_0-4h_ r^2^=0.86, P=5.15x10^-16^). There was no significant association between *ABCB1* 3435C→T and ratios of CSF-to-plasma AUC or concentration (p>0.05 for each comparison). In exploratory analyses, CSF-to-plasma ratios were not associated with 276 polymorphisms across 16 membrane transporter genes.

**Conclusions:**

Among HIV-negative adults, CSF raltegravir concentrations do not differ by *ABCB1* 3435C→T genotype but strongly correlate with plasma exposure.

**Trial Registration:**

ClinicalTrials.gov NCT00729924 http://clinicaltrials.gov/show/NCT00729924

## Introduction

The central nervous system is involved by HIV-1 early during primary infection [1,2] and 40% of patients who develop acquired immunodeficiency syndrome (AIDS) without receiving antiretroviral therapy develop symptomatic cognitive impairment [1–3]. Antiretroviral drugs vary in their ability to penetrate into the central nervous system [4], and there is some evidence that antiretroviral regimens with better central nervous system penetration are associated with better neurocognitive functioning [5]. 

The human immunodeficiency virus (HIV)-1 integrase inhibitor raltegravir is generally safe, effective and well tolerated, and is included among preferred multidrug regimens as initial HIV-1 therapy [6]. Raltegravir is metabolized primarily by glucuronidation, and is subject to relatively few drug-drug interactions. While raltegravir achieves measurable cerebrospinal fluid (CSF) concentrations, previous reports suggested that CSF-to-plasma raltegravir concentration ratios vary as much as 50-fold between individuals[7,8]. 

The ATP-binding cassette transporter superfamily genes encode ATP-ase efflux pumps involved in transport of small molecules and ions across the cell membrane. Polymorphisms in these genes are involved in the pathogenesis of cystic fibrosis, Dubin-Johnson Syndrome and Familial alpha-lipoprotein deficiency as well as several other heritable diseases[9]. One member of this gene family, the ATP-binding cassette (ABC) transporter P-glycoprotein (P-gp), encoded by *ABCB1* (previously called *MDR1*), is expressed in the blood-brain barrier and other tissues including intestine, liver, kidney, placenta, and lymphocytes where it limits tissue penetration, cellular accumulation, and promotes elimination of a variety of structurally unrelated xenobiotics [10,11]. Overexpression has been attributed to failure of CNS-targeting therapies including chemotherapeutics and anti-epileptics[12].

*ABCB1* is highly polymorphic, and a frequent variant (3435C→T, rs1045642) was in initial reports found to decrease P-gp expression in the GI tract with subsequent increase in plasma digoxin concentrations[13]. In subsequent investigations, this variant has generally[14–19], but not always[20,21] been associated with decreased P-gp function or expression. There has also be controversy regarding its effect on interindividual variability in CSF drug penetration and response, with some [22–24], but not all studies [25] showing an effect.

This polymorphism is highly prevalent in many populations. The C allele has a frequency of 43% in European populations, 55% in East Asian populations, and 90% in African populations[26]. It is a synonymous coding variant present on exon 26 of the *ABCB1*, and is not known to have an effect on either protein sequence or substrate specificity[27,28].

Raltegravir is a substrate *in vitro* for P-gp [29,30]. It is therefore plausible that functional *ABCB1* variants confer interindividual variability in raltegravir disposition into the central nervous system, lymphocytes, or other sites that harbor HIV-1. In the present study we achieved the primary goal of characterizing determinants of raltegravir disposition from plasma into CSF, and to test the hypothesis that *ABCB1* 3435C→T is associated with altered disposition. We secondarily explore other genetic variants in *ABCB1* and 15 other transporter genes expressed in the blood-brain barrier.

## Methods

The protocol for this trial and supporting TREND checklist are available as supporting information on-line; see Checklist S1 and Protocol S1.

### Study Participants

**Figure 1:**
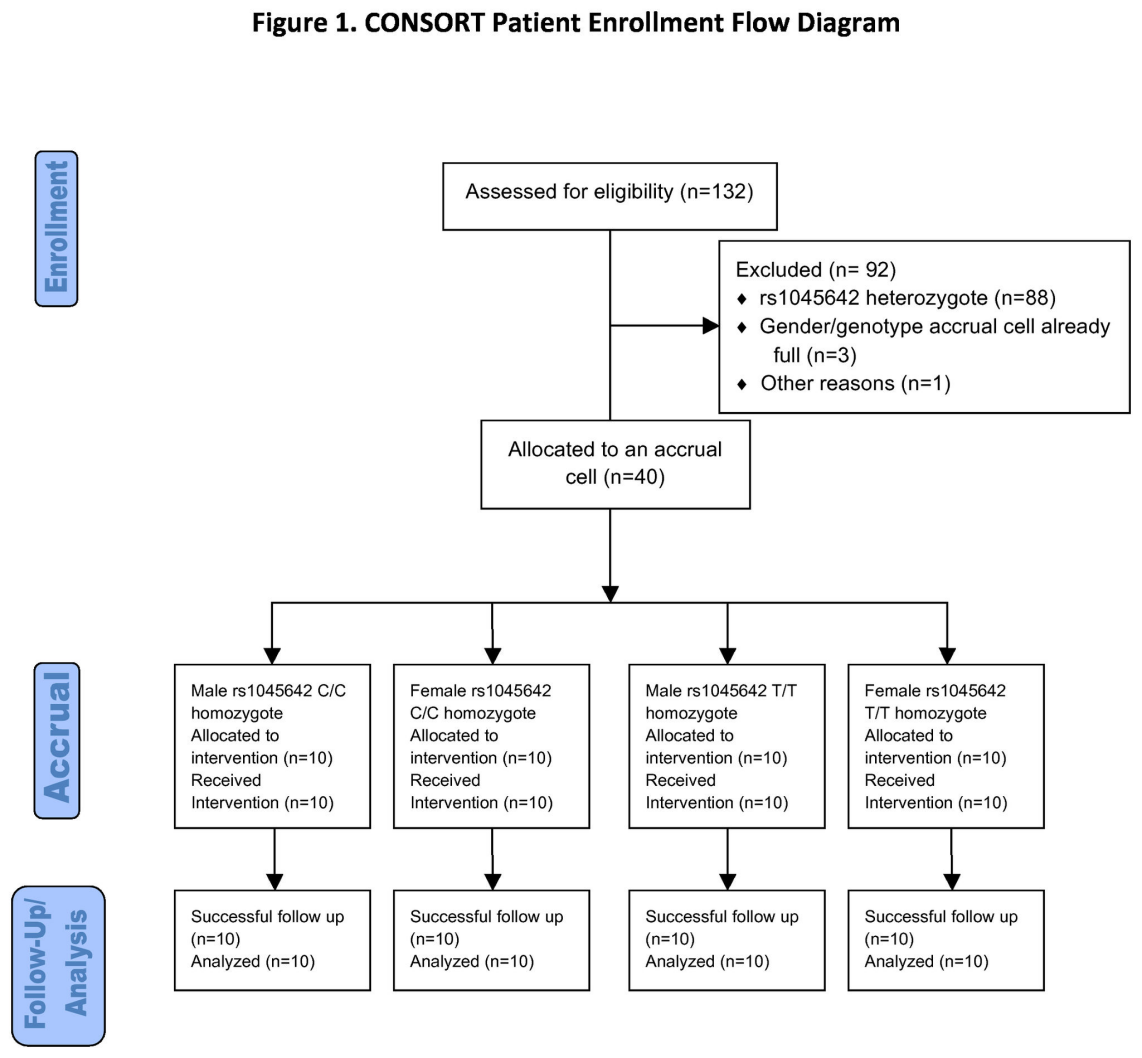
CONSORT Patient Enrollment Flow Diagram.

The study enrolled 40 healthy, HIV-negative participants, comprising 20 homozygous for *ABCB1* rs1045642 C/C (10 male and 10 female), and 20 homozygous for *ABCB1* rs1045642 T/T (10 male and 10 female) (Figure **1**). A total of 132 individuals were screened, of whom 44 were found to be homozygous at rs1045642. Three male rs1045642 C/C homozygotes and 1 female C/C homozygote were not included since 10 participants of same sex and genotype had already been accrued. One rs1045642 T/T female was removed after screening due to spinal problems. All enrolled participants completed the study. Participants were recruited from the community through email and flyers at Vanderbilt University between October 2008 and February 2011. They had acceptable screening medical history, physical exam, and laboratory findings including negative HIV-1 serology. Exclusion criteria included major medical issues, age less than 18 years or greater than 55 years, known hypersensitivity to study drug or its formulation, body mass index (BMI) > 30 kg/m^2^, pregnancy, breast feeding, receipt of medications within 14 days prior to initiating study drug known or suspected to interact substantially with P-gp, cytochrome P450 (CYP) 3A, or UDP glucuronyltransferase family 1 polypeptide A1 (UGT1A1), and central nervous system or spinal abnormality that might preclude lumbar puncture.

Homozygosity for *ABCB1* rs1045642 T/T is infrequent among individuals of African descent, and would have required screening an estimated 1100 individuals to accrue 20 homozygotes of each genotype[26]. Additionally, analysis of ethnically heterogenous populations can be confounded by population stratification[31]. We therefore limited screening to self-identified whites, defined as European descent and non-Hispanic ethnicity of the participants and both parents. At screening, only the first 10 individuals of each homozygous rs1045642 genotype of each gender (such that there were 10 males of both rs1045642 C/C and T/T genotypes, as well as 10 females of each genotype for a total of 40 individuals) participated in the pharmacokinetic component of the study. All other individuals, including all heterozygotes, did not participate in pharmacokinetic analyses, but were subsequently genotyped more extensively for other *ABCB1* variants. Authors and study personnel were blinded to both clinical and genotype data until completion of the study.

When this study was designed there were no human data regarding CSF-to-plasma raltegravir ratios. The minimum sample size to detect with 80% power and an alpha of 0.05 a difference of 1 standard deviation between *ABCB1* C/C and T/T genotype groups was 17 participants per group. Twenty participants per group were enrolled in case some were not evaluable. 

### Ethics Statement

The Vanderbilt University Institutional Review Board approved the study and all participants provided written informed consent. The study is registered with ClinicalTrials.gov (identifier NCT00729924, http://clinicaltrials.gov/show/NCT00729924). Study was performed in accordance with the Helsinki declaration of 1975.

### Genotyping assays

Eligibility genotyping for *ABCB1* rs1045642 was by TaqMan^TM^. Two MassARRAY® iPLEX Gold assays were subsequently used for exploratory analyses, one comprising 76 *ABCB1* polymorphisms, another comprising 222 polymorphisms across *ABCB1* and 15 other transporter genes expressed in the blood-brain barrier [32–34]: *ABCC1, ABCC4, ABCC5, ABCG2, SLC15A1, SLC15A2, SLC22A2, SLC22A3, SLC22A6, SLC22A8, SLCO1A2, SLCO1B1, SLCO1C1, SLCO2B1*, and *SLCO3A1*. Due to overlap between the assays there were 273 unique polymorphisms. For *ABCB1* we tagged the entire gene, including 20 kB in each 5’ and 3’ untranslated regions (UTR), using a cosmopolitan strategy, with a 5% allelic frequency cut-off, a 0.80 threshold for r^2^, 85% data convergence for tagging polymorphisms, and 70% data convergence for clustering, using all HapMap3 populations. For the 15-gene assay, polymorphisms of interest were initially gathered from PharmGKB [35], and additional polymorphisms from Ensemble [36]. Minor allele frequencies (MAF) for comparison were obtained from online databases [37,38]. We included all non-intronic polymorphisms with MAF ≥0.05 in at least one HapMap3 population, and intronic polymorphisms close to splice sites. Among polymorphisms in linkage disequilibrium (LD) at r^2^ > 0.8 in the combined group of Utah residents with ancestry from northern and western Europe (CEU), Yoruba in Ibadan, Nigeria(YRI), Japanese in Tokyo, Japan (JPT) and Han Chinese in Beijing (CHB) populations (based on HapMap release 23 modified for use with PLINK), only one polymorphisms was included in the genotyping assay. Polymorphisms that failed Sequenom assay design were replaced with tagging polymorphisms in LD at r^2^ >0.8 for either CEU or YRI populations. Polymorphisms with genotyping failure >5% were excluded. Genotyping success in the final dataset was 99.8%. Details of final Sequenom assay design are available upon request.

### Pharmacokinetics

Study participants received standard dose raltegravir 400 mg (Merck & Co., Whitehouse Station, NJ) orally every 12 hours for 7 days (14 doses) to achieve steady-state plasma concentrations. Participants kept medication diaries to record dose times and missed doses. Participants were admitted to the Vanderbilt Clinical Research Center 13 hours prior to the final dose. Research staff administered the penultimate dose under direct observation 12 hours prior to the final dose, immediately following a small meal, and also administered the final dose. Participants fasted for 8 hours before the final dose and for 3 hours thereafter. In all participants, plasma for raltegravir assay was obtained 15 minutes before the final dose, and again 1, 2, 3, 4, 5, 6, 8, and 12 hours post-dose. For plasma raltegravir assays, whole blood was collected in 10 mL EDTA tubes, immediately placed on ice, and centrifuged within 1 hour at 800 x *g* and 4°C for 10 minutes. In the final 20 participants, additional plasma specimens for raltegravir assay were obtained 16, 20, 24 and 48 hours after final dose. In all participants, lumbar puncture to obtain 15 mL of CSF was performed 4 hours after final dose. The CSF was immediately placed on ice, aliquots removed for total protein and cell count assays, and the remaining CSF centrifuged within 10 minutes at 800 x *g* at 4°C for 10 minutes. Plasma and cell-free CSF supernatant were aliquotted and stored at -80^O^C until assayed. Concentrations of raltegravir in plasma and CSF were quantitated using a validated LC/MS/MS assay with a dynamic range of 10-10,000 ng/mL, as described elsewhere [39]. Precision over the calibration range was within 1.9% to 9.9%, while accuracy for each calibration standard range from -5.6% to 3.9%. Using a calibration curve prepared in K3EDTA, overall inter-day precision (% coefficient of variation (CV)) of lower limit of quantitation, low, mid, and high quality controls were 5.8%, 2.0%, 4.0%, and 2.5%, while overall accuracies (% deviation) were 3.3%, -3.4%, -2.8%, and -0.3%, respectively. Precision (% CV) of matrix-matched lower limit of quantitation, low, mid, and high quality controls prepared in K3EDTA ranged from 2.2% to 8.8%, 1.2% to 2.5%, 1.1% to 2.2%, and 0.8% to 1.6%, while accuracies (% deviation) ranged from 0.8% to 6.2%, -3.6% to -3.1%, -5.5% to 2.2%, and -2.0% to 2.7%, respectively.

### Derivation of pharmacokinetic parameters

Plasma pharmacokinetic parameter estimates were determined using a non-compartmental approach (WinNonlin version 4.01, Pharsight Corp., Mountain View, CA.). Area under the concentration-time curve (AUC) was determined using the linear/log trapezoidal method. Maximum plasma concentration (C_max_), time to C_max_ (T_max_), and plasma concentration at each timepoint were taken directly from the observed concentration-time data. Oral clearance (CL/F) was calculated as dose/AUC_0-12h_. Apparent volume of distribution (V_z_/F) was calculated as dose divided by the product of the elimination rate constant (λ_z_) and AUC_0-12h_. The elimination rate constant was determined by linear regression of the terminal elimination phase concentration-time points. Elimination half-life (t_1/2_) was calculated as ln(2)/λ_z_. Assay results below limit of quantification were treated as 5.0 ng/mL. The raltegravir 95% inhibitory concentration (IC)_95_ is 31 nM ± 20 nM (13.8 ng/mL ± 8.9 ng/mL) for human T-lymphoid cell cultures infected with the cell-line adapted HIV-1 variant H9IIIB, and ranged from 9 nM to 19 nM (4.0 ng/mL to 8.5 ng/mL) based on cultures of 5 clinical HIV-1 subtype B isolates in mitogen-activated human peripheral blood mononuclear cells (without adjusting for protein binding) [40]. 

### Statistical analyses

Statistical analyses involving genetic data were performed with PLINK version 1.07. For association analyses with individual polymorphisms logistic regression and Wald test were used to derive, with max(T) permutation used to empirically derive P value thresholds for multiple testing [41,42]. For polymorphisms in complete linkage disequilibrium in our study cohort, only one was retained in the analyses. Polymorphisms lacking minor alleles were excluded. Raltegravir AUC and concentration values were log transformed for statistical analysis. Ratios of raltegravir AUC and/or concentration values were based on non-log transformed values. Statistical analysis of non-genetic variables was done with the R statistical software package version 2.13.0[43]. All analyses were corrected for age, gender and BMI. Analyses involving CSF raltegravir concentrations were also corrected for CSF protein concentration.

## Results

### Participant characteristics

A total of 132 individuals underwent *ABCB1* rs1045642 genetic screening for study eligibility, of whom 40 (20 homozygous for C/C, 20 homozygous for T/T, each group split equally between males and females) were selected for study participation, initiated raltegravir, and underwent pharmacokinetic analyses. Among the 40 participants, the median age was 34.5 years, females were older than males with a median age of 39.0 versus 28.0 years. Median BMI in men was 23.6 kg/m^2^ versus 24.7 kg/m^2^ in females, and median weight was 75 kg. Total protein in CSF was significantly higher in males, as reported elsewhere [44] (Table **1**).

**Table 1:** Participant characteristics and raltegravir pharmacokinetic parameters.

**Parameter**	**Males**	**Females**	**All**	**P value**
	**Median (IQR)**	**Median (IQR)**	**Median (IQR)**	
	**n=20**	**n=20**	**n=40**	
**Baseline Characteristics**				
Age (years)	28.0 (25.8 - 37.5)	39.0 (33.3 - 47.8)	34.5 (26.8 - 45.3)	**0.024**
Weight (kg)	79.8 (75.6 - 84.4)	64.1 (57.6 - 70.7)	74.6 (62.6 - 80.5)	**<0.001**
BMI (kg/m^2^)	23.6 (22.8 - 25.5)	24.7 (21.3 - 25.8)	24.0 (22.2 - 25.6)	0.88
CSF protein **^*a*^**	42.5 (33 - 44.25)	24.0 (21 - 32.3)	33.0 (24 - 43)	**<0.001**
**Pharmacokinetics**				
T_½_ (h)	4.1 (3.2 - 12.0)	7.1 (3.9 -14.5)	5.5 (3.5-13.4)	0.19
Time Above IC_95_ After 12 hours (h)	1.7 (-0.1 - 9.9)	5.5 (-1.2 - 11.4)	3.0 (-0.4 - 10.8)	0.68
T_max_ (h)	3.0 (2.0 - 4.0)	2.5 (1.0 - 3.0)	3.0 (2.0 - 3.9)	0.11
V/F (L)	465 (183 - 860)	807 (239 - 1652)	540 (201 - 1388)	0.23
CL/F (L/h)	57.9 (33.1 - 77.4)	56.6 (28.3 - 222.1)	57.9 (30.7 - 114.7)	0.78
C_max_ **^*b*^** (ng/mL)	2228 (1396 - 4018)	1679 (541 - 5808)	1932 (1038 - 4560)	0.66
4 hour plasma concentration **^*b*^** (ng/mL)	1452 (865 - 2355)	820 (270 -1750)	1091 (710 - 2218)	0.072
4 hour CSF concentration ^***a***,***b***^ (ng/mL)	28.6 (19.1 - 46.2)	31.5(15.6 - 66.1)	30.1 (17.0 - 50.4)	0.55
Plasma AUC_0->12_ (h*mg/L) **^*b*^**	7.26 (5.17 - 12.1)	5.92 (1.80 -14.2)	6.55 (3.48 - 13.03)	0.77
12 hour plasma concentration (ng/mL) **^*b*^**	47.2 (32.1 - 66.8)	43.3 (27.8 - 69.0)	45.2 (31.2 - 66.8)	> 0.99
Ratio of CSF concentration to 2 hour plasma concentration	0.038 (0.020-0.061)	0.028 (0.019-0.044)	0.033 (0.019 - 0.049)	0.27
Ratio of CSF concentration to plasma AUC_0->4_ (h)	0.0063 (0.0056 - 0.0073)	0.0073 (0.0062 - 0.010)	0.0068 (0.0059 - 0.0080)	0.13

^a^ – Abbreviations: CSF cerebrospinal fluid

^b^ – Geometric mean(IQR)

### Plasma raltegravir pharmacokinetics

**Figure 2:**
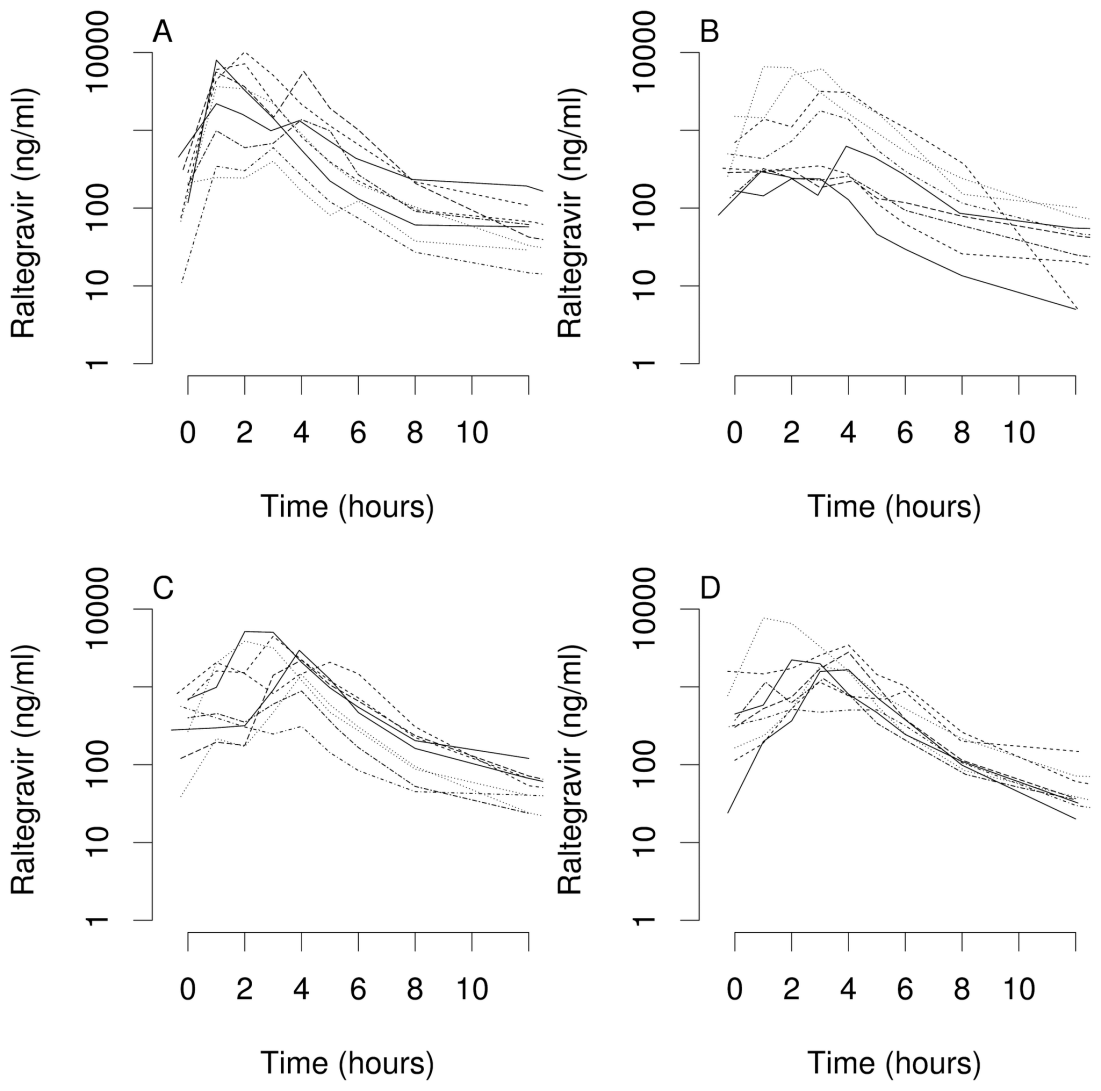
Plasma raltegravir concentration-time profiles. Panels represent plasma raltegravir concentration-time profiles from pre-dose to 12 hours post-dose for all 40 participants. Participants are stratified as homozygous for rs1045642 C/C (Panels A and C), rs1045642 T/T (Panels B and D), females (Panels A and B), and males (Panels C and D).

All 40 participants underwent plasma pharmacokinetic sampling for 12 hours after the final dose, and 20 continued sampling to 48 hours after the final dose. Plasma pharmacokinetic parameters are shown in Table **1**. Plasma concentration-time profiles for participants (0 to 12 hours), stratified by sex and *ABCB1* genotype, are shown in Figure **2**. Mean plasma T_max_ was 2.7 hours (IQR 2.0 to 3.9 hours). The geometric mean concentration at 12 hours and AUC_0-12h_ were 45.2 ng/mL (IQR 31.2 to 66.8 ng/mL, range <10 to 243.1 ng/mL) and 6.55 h·mg/L (IQR 3.48 to 13.03 h·mg/L, range 0.95 to 24.33 h·mg/L), respectively, similar to previously reported values of 68.5 ng/mL and 6.35 h·mg/L [40]. Among the 20 individuals who continued sampling to 48 hours, the 48 hour geometric mean concentration was 7.47 ng/mL, however 14 participants had concentrations below limit of quantification, which were treated as 5 ng/mL. None of the plasma pharmacokinetic parameters differed significantly by sex, BMI, rs1045642 genotype or other polymorphism genotype (p > 0.05 for each comparison).

### Correlations between CSF and plasma raltegravir exposure

**Figure 3:**
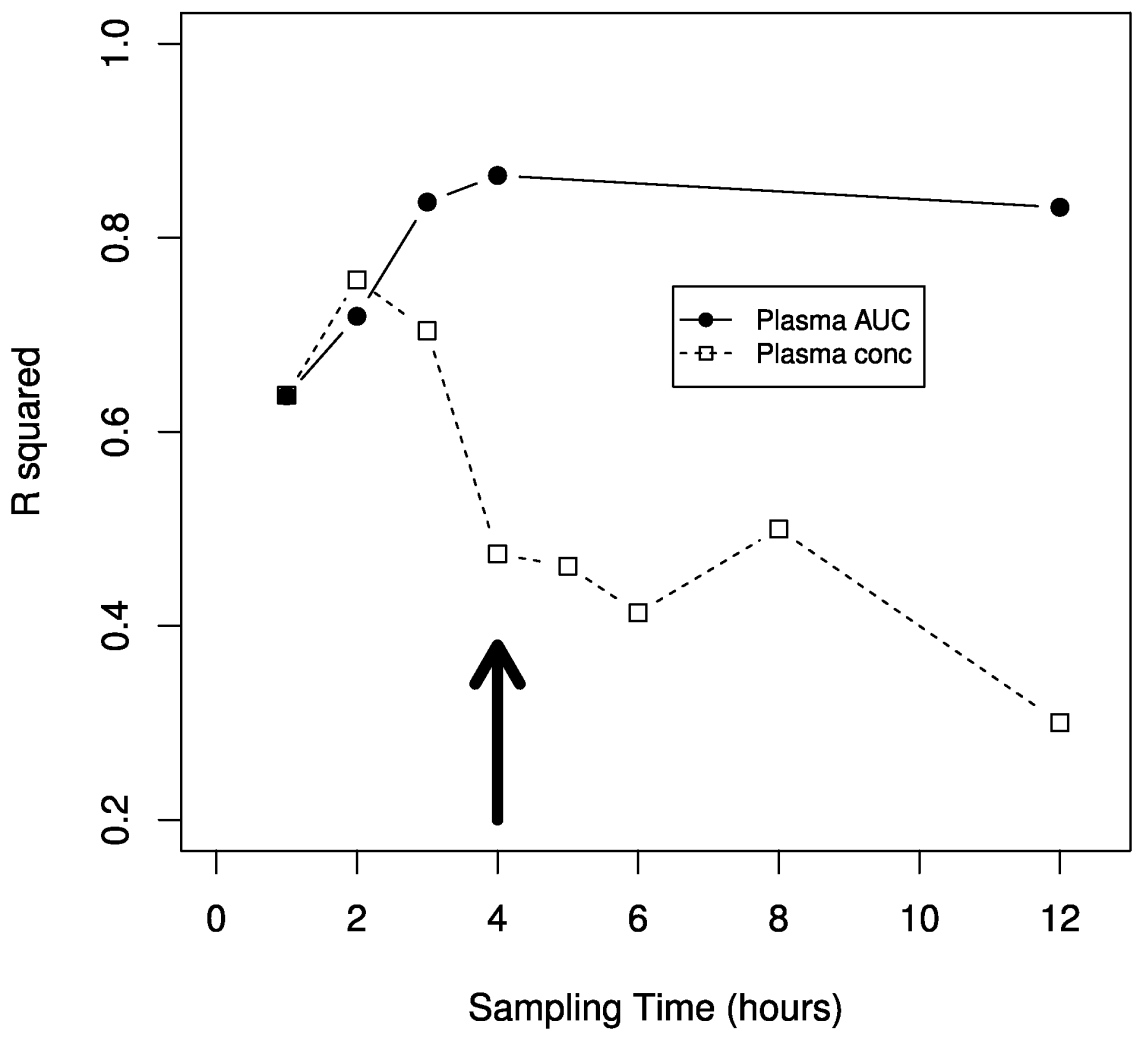
Correlations between 4-hour post-dose raltegravir CSF concentrations, plasma partial AUC values, and single timepoint plasma concentrations. Curves represent correlations (measured by linear regression r^2^) between 4-hour post-dose CSF raltegravir concentrations and plasma raltegravir parameters. Correlations with plasma raltegravir partial AUC values from time 0 to each post-dose timepoint (solid line, solid circles). Correlations with single timepoint plasma raltegravir concentrations each at post-dose timepoint (dashed line, open squares). Bold vertical arrow indicates time of CSF collection.

All 40 participants had CSF obtained 4 hours after the final dose. There was an approximately 25-fold inter-individual range in CSF raltegravir concentrations at 4 hours post-dose (geometric mean 30.1 ng/ml, IQR 17.0 to 50.4 ng/ml, range ≤10 to 130 ng/ml) (Table **1**). Median CSF raltegravir concentrations did not differ significantly between females and males (p = 0.55), and CSF raltegravir concentrations did not correlate significantly with CSF total protein (p > 0.99). We next identified which index of plasma raltegravir exposure best correlated with 4-hour CSF raltegravir concentrations (adjusting for age, sex, BMI and CSF protein concentration). Among partial plasma AUCs, the partial plasma AUC from 0 to 4 hours (AUC_0-4h_) correlated most strongly with 4-hour CSF concentrations (r^2^ = 0.86, p = 5.15 x 10^-16^), although all partial AUCs beyond 2 hours were also strongly correlated with 4-hour CSF concentrations. Among single timepoint plasma concentrations, the 2-hour value correlated most strongly with 4-hour CSF concentrations (r^2^ = 0.76, p = 1.12 x 10^-11^). The correlation between the 4-hour CSF concentration and the simultaneous 4-hour plasma concentration was considerably less (r^2^ = 0.47, p=6.89 x 10^-6^, Figure **3**). 

Cerebrospinal fluid is a slow equilibrating compartment compared to plasma. Ratios of CSF-to-plasma drug concentrations therefore vary depending on CSF sampling timing, and are generally highest later in the dosing interval. In the present study the mean ratio of 4 hour CSF concentration to mean 0-12 hour plasma raltegravir concentrations in each participant was 6.0% ± 2.6% (mean ± SD).

### Relationship between *ABCB1* genotype and raltegravir CSF-to-plasma ratios

**Figure 4:**
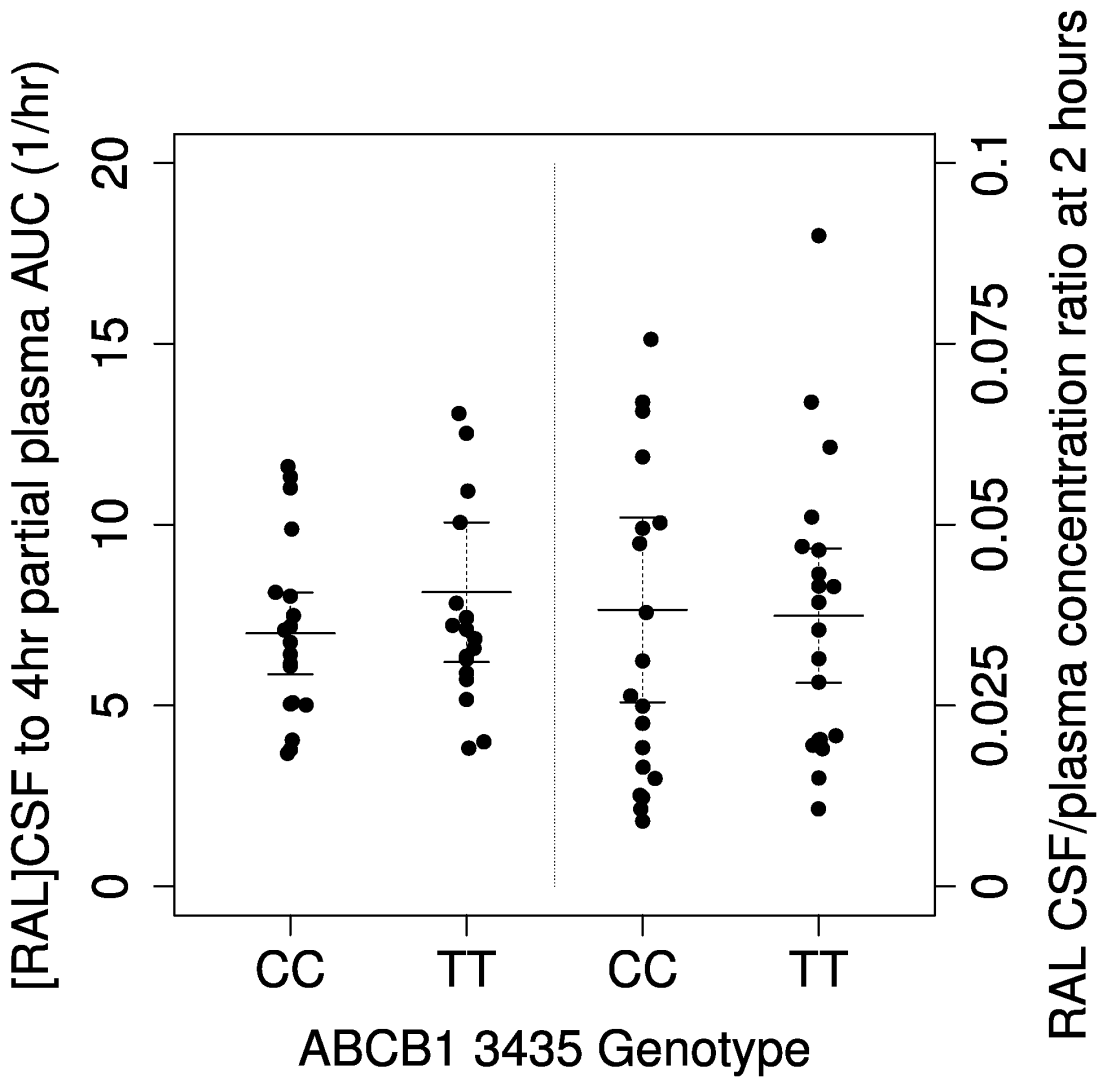
Relationship between *ABCB1* rs1045642 genotype and CSF-to-plasma raltegravir ratios. Ratio of 4-hour post-dose CSF raltegravir concentration to selected plasma raltegravir parameters stratified by *ABCB1* 3435 genotype. The left half of figure represents ratios of 4-hour post-dose CSF raltegravir concentrations to 0-4 hour partial plasma raltegravir AUC values; the right half of figure represents ratios of 4-hour post-dose CSF raltegravir concentrations to 2-hour post-dose plasma raltegravir concentrations. Plasma concentrations below the limit of detection were considered to be 5.0 ng/ml for this figure. Error bars represent 95% CI.

Plasma raltegravir AUC_0-4h_ explained 86% of interindividual variability in 4-hour CSF raltegravir concentrations, as noted above. To assess the contribution, if any, of *ABCB1* rs1045642 genotype we compared CSF-to-plasma ratios by genotype based on plasma AUC_0-4h_ values and based on the 2-hour plasma concentration timepoint. Neither CSF-to-AUC_0-4h_ ratio (p=0.28, β=-0.18, 95% CI (-0.50, 0.14)) nor CSF-to-2-hour plasma ratio (p=0.73, β= -0.060, 95%CI (-0.40, 0.27)) differed significantly among individuals homozygous for *ABCB1* rs1045642 C/C versus T/T (Figure **4**). No significant association was detected between absolute CSF raltegravir concentrations (regardless of plasma concentrations) and *ABCB1* rs1045642 genotype (data not shown). 

### Exploratory analyses with additional genetic variants

A total of 269 genetic polymorphisms passed QC, of which 117 were censored for either being monomorphic, or being in complete LD (r^2^ =1.0) with at least one other polymorphism in our study cohort. The remaining 152 polymorphisms across 16 transporter genes were explored for association with raltegravir penetration into CSF, using the same approach as for *ABCB1* rs1045642. Ten of the 152 polymorphisms (not including rs1045642) had Harvey Weinberg equilibrium P values less than 0.05, while two polymorphisms differed from MAF proportions for populations of European descent in public databases at P values less than 0.05 (rs9457880, P = 0.017; rs4149179, P = 0.043) [38]. Among the 40 participants with CSF data, at a nominal P value threshold of 0.05, 5 polymorphisms were associated with CSF-to-plasma ratios based on the 2-hour plasma timepoint, and 6 based on plasma AUC_0-4h_ values (Table **2**). None were significant at a conservative Bonferroni-corrected P-value threshold of 3.3 x 10^-4^ (152 comparisons), or by empirically derived permutation adjusted P values. A list of polymorphisms, minor allele frequencies, beta (with confidence intervals), and P-values for association with CSF-to-plasma AUC_0-4h_ ratios is provided in on-line Supplemental Material (Table **S1**). 

**Table 2:** SNPs associated with ratio of CSF-to-plasma raltegravir concentration and exposure.

Chromosome			SNP	P value	Associated Gene
		15	rs9302356	0.05	*SLC03A1*
		15	rs7496880	0.047	*SLC03A1*
		13	rs57270423	0.046	*ABCC4*
		13	rs1517618	0.024	*ABCC4*
		15	rs1289389	0.01	*SLC03A1*
		16	rs3784862	0.005	*ABCC1*
		12	rs11045919	0.05	*SLCO1A2*
		6	rs2076828	0.039	*SLC22A3*
		3	rs3805114	0.035	*ABCC5*
		6	rs2450975	0.031	*SLC22A2*
		6	rs316003	0.026	*SLC22A2*

CSF concentration / plasma AUC_0->4_

CSF concentration / plasma concentration at 2 hours

### Adverse events

One participant experienced a post-lumbar puncture headache that resolved with placement of an epidural blood patch. There were otherwise no grade 3 or higher adverse events.

## Discussion

The central nervous system is involved by HIV-1 throughout the course of infection [1,2], and antiretroviral drugs that penetrate more effectively may have neurocognitive benefits [5]. Raltegravir is a substrate for P-gp [29,30], an ATP-dependent drug-efflux transporter expressed in the blood-brain barrier. This study is the first to prospectively characterize genetic and non-genetic correlates of raltegravir CSF penetration with intensive pharmacokinetic sampling and pharmacogenetic analysis. Additionally, the study is designed specifically to investigate the association of raltegravir CSF penetration with a common functional *ABCB1* polymorphism. Several aspects of the present study design were novel compared with previous studies. This allowed us to demonstrate that plasma raltegravir exposure explained the majority (86%) of interindividual variability in 4-hour CSF raltegravir concentrations, and that the *ABCB1* rs1045642 variant did not contribute significantly to interindividual variability in CSF-to-plasma raltegravir ratios.

In the present study, there was marked inter-individual variability in the range of plasma raltegravir pharmacokinetic parameters and CSF raltegravir concentrations (e.g. 45-fold for 4-hour plasma raltegravir concentrations and 27-fold for 4 hour CSF raltegravir concentration). In contrast, the range of CSF-to-2-hour-plasma raltegravir ratios varied only 12-fold, and the interquartile range varying about the median by less than 50% (and by less than 20% for the CSF-to-AUC_0-4h_ ratio). This differs with two previous studies in which the range of CSF-to-plasma raltegravir concentration ratios varied as much as 50-fold between individuals [7,8]. The difference between the present and previous studies likely reflects different sampling strategies and study populations. In a study of 25 paired plasma and CSF samples from 16 HIV-infected patients on raltegravir-containing regimens, Yilmaz et al found no significant correlation between plasma and CSF raltegravir concentrations [8]. An association was seen between CSF raltegravir concentrations and both CSF albumin and CSF-to-plasma albumin ratios, perhaps reflecting decreased blood-brain barrier integrity. In a separate study involving 21 paired plasma and CSF samples from 18 HIV-infected patients on raltegravir-containing regimens, Croteau et al found only a weak correlation between CSF and plasma raltegravir concentrations (*r* ^2^ = 0.24, P = 0.02)[7]. In both studies time from previous raltegravir dose to paired CSF and plasma sampling varied considerably among individuals. Cerebrospinal fluid is a slow equilibrating compartment (i.e. peak drug concentrations occur later in CSF than in plasma, and differences between peak and minimum drug concentrations are less in CSF than in plasma) [45,46]. By collecting CSF at the same timepoint in all participants we minimized confounding from time-dependent variation in CSF-to-plasma ratios. By serially quantifying plasma raltegravir concentration at multiple timepoints we could empirically determine which plasma exposure variable(s) most strongly correlated with CSF concentrations. In this regard, the P-value for association with the 4-hour plasma sample (P =6.89 10^-6^, r^2^=0.47) was approximately 6 orders of magnitude greater than that for the 2-hour plasma sample (P = 1.12 10^-11^, r^2^=0.76), and approximately 10 orders of magnitude greater than that for the plasma AUC_0-4h_ (P = 5.15 10^-16^, r^2^=0.86). By studying healthy participants we minimized potential confounding by altered blood-brain barrier integrity. This study design may be adapted to efficiently characterize CSF disposition of other antiretroviral drugs.

The multidrug transporter P-gp is expressed in the blood-brain barrier and various other tissues [10,11]. In 2000 Hoffmeyer et al stimulated considerable interest by implicating the 3435 T allele with lower expression of P-gp in the gut and increased plasma digoxin concentrations [13]. A subsequent report suggested that this synonymous polymorphism (i.e. resides in an isoleucine codon but does not change the residue) might affect P-gp structure and function by altering protein folding and membrane insertion [16]. However, efforts to replicate associations with *ABCB1* 3435C→T have yielded inconsistent results. For example, regarding ABCB1 expression, 3435 T has been associated with decreased [14–19], increased [20], or no effect [21]. Regarding pharmacokinetics of various drugs, 3435 T has been associated with increased [19,47,48], decreased [49,50], or no effect [51–53] on exposure. Regarding the treatment of epilepsy (an example of CNS-targeted therapy) 3435 T has been associated with drug susceptibility [23,24,54], drug resistance [55,56], or no effect [25]. Such inconsistencies suggest either spurious associations, or that complexities of *ABCB1* genotype-phenotype associations have yet to be defined. 

The present study was designed primarily to assess associations with *ABCB1* 3435C→T. However, raltegravir is also a substrate for the solute carrier (SLC) transporters SLC22A6 and SLC15A1 [29,30]. In exploratory analyses involving 272 additional polymorphisms in *ABCB1* and 15 other transporter genes, we found no significant association between raltegravir CSF-to-plasma ratios and any of these additional polymorphisms, including 85 in *ABCB1*, 2 in *SLC22A6*, and 8 in *SLC15A1*. However, minor allele frequencies for many of these polymorphisms were low in our cohort, and only 209 polymorphisms had at least one individual homozygous for the minor allele. Additionally, 55 polymorphisms were redundant due to complete linkage disequilibrium in our population. Confidence intervals for associations with transporter polymorphism genotypes were large, in part due to the low minor allele frequencies, indicating relatively low power to detect true associations for these additional polymorphisms (see Table **S1**). 

There were limitations to this study. While we explored associations between CSF-to-plasma raltegravir ratios and many transporter gene variants, this study had greatest power to assess *ABCB1* 3435C→T, and may have not had sufficient power to detect associations with other polymorphisms assayed. Additionally, smaller differences in CSF-to-plasma concentration ratios between the two *ABCB1* 3435C genotypes may not have been found. We cannot exclude the possibility that transporter gene variants affect raltegravir disposition into CSF. When this study was designed we anticipated that CSF raltegravir concentrations might be extremely low. We therefore sampled CSF at 4 hours to approximate peak plasma concentrations and increase the likelihood of quantifiable CSF concentrations. It is possible that effects of transporter gene variants will be more apparent with CSF obtained later than 4 hours post-dose, particularly if plasma raltegravir exposure explains considerably less than 86% of interindividual variability in CSF raltegravir concentrations at later timepoints. While the best predictors of 4-hour post-dose CSF raltegravir concentrations were 2-hour post-dose plasma concentrations and plasma AUC_0-4h_ values, such plasma-CSF relationships may differ for other drugs. Additionally, we did not account for interindividual variation in low level immune activation which may be associated with variation in blood brain barrier integrity. It is possible that raltegravir concentrations differ in CSF versus brain parenchyma, or that modulation of P-gp activity through non-genetic means such as P-gp inhibitors affects raltegravir pharmacokinetics but we know of no data that address these questions. 

In summary, in this cohort of healthy, HIV-negative volunteers dosed to steady state with twice-daily raltegravir, 4-hour post-dose CSF raltegravir concentrations are determined primarily by plasma drug exposure, and were not affected by the *ABCB1* 3435C→T variant. This study design is well suited to quantify the impact of specific blood-brain barrier transporter gene variants on drug disposition into CSF.

## Supporting Information

Checklist S1 **TREND Checklist.**
(DOC)

Protocol S1 **Trial Protocol.**
(DOC)

Table S1 **Listing of all polymorphisms assayed, minor allele frequencies, and beta and P-values for association with CSF-to-plasma AUC_0-4h_ ratios in study participants.**
(DOC)
