# Supplementary material for: Genetic and Non-Genetic Determinants of Raltegravir Penetration into Cerebrospinal Fluid: A Single Arm Pharmacokinetic Study
Source: PLoS One. 2013 Dec 11;8(12):e82672. doi: 10.1371/journal.pone.0082672 (PMC3859605; doi:10.1371/journal.pone.0082672)
Supplement: Protocol S1 — Trial Protocol. (DOC) [file pone.0082672.s002.doc]

**Genetic Predictors of Raltegravir Penetration into Cerebrospinal Fluid**

(This is an investigator-initiated study.)

## Principal Investigator:

## David W. Haas, M.D.

Associate Professor of Medicine,

Microbiology, and Immunology

Vanderbilt University School of Medicine

Director, Vanderbilt AIDS Clinical Trials Center

345 24th Avenue North, Suite 105

Nashville, TN 37203

**Table of Contents:**

**Study Schema**

1. **Background**
2. **Rationale and Specific Aims**
3. **Animal Studies and Previous Human Studies**
4. **Inclusion/Exclusion Criteria**
5. **Enrollment/Randomization**
6. **Study Procedures**
7. **Risks of Investigational Agents/Devices (side effects)**
8. **Reporting of Adverse Events or Unanticipated Problems involving Risk to Participants or Others**
9. **Study Withdrawal/Discontinuation**
10. **Statistical Considerations**
11. **Privacy/Confidentiality Issues**
12. **Follow-up and Record Retention**

**APPENDICES**

**Appendix A:** Schedule of events

**Appendix B:** References

**Appendix C:** Laboratory Processing Chart

**Appendix D:** Upper Limit Weight Cut-offs for Study Participation

# Appendix E: Pharmacokinetic (PK) Evaluations

**SCHEMA**

**Genetic Predictors of Raltegravir Penetration into Cerebrospinal Fluid**

DESIGN: This single arm, open-label healthy volunteer study will determine whether there are associations between a frequent C>T single nucleotide polymorphism (SNP) at position 3435 (rs1045642) in the drug transporter gene *ABCB1* (also known as *MDR1*) and penetration of raltegravir (RGV) into cerebrospinal fluid (CSF). Study participants will take RGV 400 mg orally every 12 hours for 7 days. On Day 7 of RGV participants will undergo 24-hour serial plasma sampling to determine steady-state plasma pharmacokinetic (PK) parameters for RGV. A plasma sample will also be obtained on Day 9. Peripheral blood mononuclear cells (PBMCs) will be obtained to measure intracellular RGV. Lumbar puncture will be performed 4 hours after the morning dose on Day 7 to quantify the CSF RGV concentration. Primary outcomes of interest will be ratios of the CSF RGV C_4h_ value to the plasma RGV C_max_, C_min_ and AUC_0-12h_ values.

DURATION: Subject’s participation may last as many as 12 months, but in most cases approximately 3 month.

SAMPLE SIZE: This study will enroll 40 subjects over approximately 2 year.

POPULATION: Healthy, HIV-negative volunteers who are at least 18 years of age, with body mass index less than 30, and with acceptable screening laboratories. Only individuals of self-reported white European ancestry, and whose parents’ were both of white European ancestry may participate. With regard to *ABCB1* 3435 C>T, the study will include 20 C/C homozygotes and 20 T/T homozygotes. Each genotype group will comprise 10 males and 10 females, as follows:

|  | *ABCB1* 3435 genotype | |
| --- | --- | --- |
|  | C/C | T/T |
| Male | 10 | 10 |
| Female | 10 | 10 |

RANDOMIZATION: None

REGIMEN: Study participants will receive RGV 400 mg orally every 12 hours for 7 days.

1. **Background**

Many antiretroviral drugs are substrates for P-glycoprotein (P-gp) [1-6]. This multidrug efflux transporter is expressed in the blood-brain barrier where it limits entry of substrate drugs into the central nervous system (CNS) [3]. Emerging data support the potential relevance of CNS drug penetration in at least some individuals [7]. Raltegravir (MK-0518) is a substrate for P-gp based on its directional transport in the well-established LLC-PK1 cell culture model. Preclinical data also indicate low brain-to-plasma concentration ratios for RGV. Further studies are warranted to elucidate the relevance of P-gp transport for RGV in the CNS.

Raltegravir has a low likelihood of drug-drug pharmacokinetic interactions (Isentress Prescribing Information, 5/2008). Raltegravir does not inhibit CYP1A2, CYP2B6, CYP2C8, CYP2C9, CYP2C19, CYP2D6 or CYP3A *in vitro*. Moreover, *in vitro*, raltegravir did not induce CYP3A4. A midazolam drug interaction study confirmed the low propensity of raltegravir to alter the pharmacokinetics of agents metabolized by CYP3A4 *in vivo* by demonstrating a lack of effect of raltegravir on the pharmacokinetics of midazolam, a sensitive CYP3A4 substrate. Similarly, raltegravir is not an inhibitor of the UDP glucuronosyltransferases (UGT) tested (UGT1A1, UGT2B7), and raltegravir does not inhibit P-glycoprotein-mediated transport. Based on these data, raltegravir is not expected to affect the pharmacokinetics of drugs that are substrates of these enzymes or P-glycoprotein. Raltegravir is not a substrate of cytochrome P450 (CYP) enzymes. Based on *in vivo* and *in vitro* studies, raltegravir is eliminated mainly by metabolism via a UGT1A1-mediated glucuronidation pathway. Rifampin, a strong inducer of UGT1A1, reduces plasma concentrations of raltegravir. Therefore, caution should be used when coadministering raltegravir with rifampin or other strong inducers of UGT1A1. Other less strong inducers may be used with the recommended dose of raltegravir. Atazanavir, a strong inhibitor of UGT1A1, and atazanavir/ritonavir increase plasma concentrations of raltegravir. However, concomitant use of raltegravir and atazanavir/ritonavir did not result in a unique safety signal in Protocol 005 and Protocols 018 and 019. Based on these data, atazanavir/ritonavir may be coadministered with raltegravir without dose adjustment of raltegravir. Coadministration of raltegravir with other drugs that inhibit UGT1A1 may increase plasma levels of raltegravir.

P-glycoprotein is encoded by *ABCB1* (previously called *MDR1*). *ABCB1* is highly polymorphic, and a frequent *ABCB1* polymorphism (3435C>T; rs1045642) has been associated with decreased P-glycoprotein activity in numerous studies [8,9]. This polymorphism is present in approximately 50% of European Americans and 17% of African Americans [10]. A number of studies (including several from our group) have suggested associations between *ABCB1* 3435C>T and responses to HIV therapy [10-14], although study results are somewhat conflicting. Some studies also suggest that another *ABCB1* polymorphism (2677G>T/A; rs2032582) which is in incomplete linkage with 3435C>T may also be associated with altered P-glycoprotein activity, although results have also been conflicting [8].

Important aspects regarding the PK of RGV have yet to be defined. Because the target of RGV is intracellular, prolonged intracellular exposure with RGV may help explain its efficacy, but the terminal elimination PK of RGV from within PBMCs among individuals who have been dosed to steady-state have not been defined. Similarly, the terminal elimination PK of RGV from plasma among individuals who have been dosed to steady-state have not been defined.

Our group has devoted considerable effort to elucidating the relevance of human genetic variability for antiretroviral drug disposition, efficacy, and toxicity [10,12-20]. The proposing investigator also has considerable experience studying HIV disease in the CNS, including pharmacokinetics of drug penetration into CSF [21-26]. The present proposal extends this work to RGV, a major addition to our therapeutic armamentarium.

1. **Rationale and Specific Aims**

Our hypothesis is that a frequent *ABCB1* single nucleotide polymorphism (SNP) influences disposition of RGV into the CNS, which may in turn be relevant for HIV treatment response in this compartment.

**SPECIFIC AIMS:**

2.0.1 **Primary aim**

1. To determine whether *ABCB1* 3435C>T is associated with altered cerebrospinal fluid (CSF)-to-plasma RGV concentration ratios among healthy volunteers at steady state.

2.0.2 **Secondary aims**

1. To determine whether *ABCB1* 2677G>T and/or other *ABCB1* SNPs are associated with altered CSF-to-plasma RGV concentration ratios among healthy volunteers at steady state.

2. To characterize PBMC intracellular pharmacokinetics of RGV at steady state, and associations with *ABCB1* 3435C>T and 2677G>T.

3. To characterize plasma terminal elimination pharmacokinetics of RGV, and associations with *ABCB1* 3435C>T and 2677G>T.

4. To store leftover DNA, plasma and CSF specimens that may be used for future unplanned analyses of RGV and/or its metabolites, and of additional human DNA polymorphisms.

1. **Animal Studies and Previous Human Studies**

See Investigator Brochure for Raltegravir (MK-0518).

1. **Inclusion/Exclusion Criteria**

4.0.1 **Study participants**

The study will enroll 40 healthy volunteers. Of these 40 individuals, 20 will be *ABCB1* position 3435 C/C homozygotes, and 20 will be 3435 T/T homozygotes. Each of the two genotype groups we comprise 10 males and 10 females. There will therefore be 4 separate groups.

4.0.2 **Inclusion Criteria**

1. Able and willing to give written informed consent.

2. Negative HIV-1 serology documented by any licensed ELISA test kit within 30 days prior to initiating study drug.

3. Men and women at least 18 but no more than 55 years of age at study entry.

4. Body mass index less than 30.

Body mass index = (weight in kilograms)/(height in meters)^2^

OR

Body mass index = [(weight in pounds)/(height in inches)^2^] X 703

5. Estimated creatinine clearance ≥ 50 mL/minute within 30 days prior to study entry, calculated by the Cockcroft-Gault method as follows:

Men: creatinine clearance = [(140 - age) x (weight in kg)]/(72 x serum creatinine)

Women: creatinine clearance = 0.85 x [(140 - age) x (weight in kg)]/(72 x serum creatinine)

6. Laboratory values obtained within 30 days prior to study entry:

- Absolute neutrophil count (ANC) ≥ 1,000/mm^3^.
- Hemoglobin ≥ 12.5 g/dL for males and ≥ 11.5 g/dL for females.
- Platelet count ≥ 100,000/mm^3^.
- AST (SGOT), ALT (SGPT), and total bilirubin within normal range.
- Alkaline phosphatase ≤ 1.5 × upper limit of normal (ULN).

7. Female study volunteers of reproductive potential (defined as women who have not been post-menopausal for at least 24 consecutive months, i.e., who have had menses within the preceding 24 months, or have not undergone surgical steriliza­tion [e.g., hysterectomy, bilateral oophorectomy, or salpingotomy]) must have a negative serum or urine pregnancy test performed within 30 days before study entry.

8. All study volunteers must agree not to participate in a conception process (e.g., active attempt to become pregnant or to impregnate, sperm donation, in vitro fertilization).

If participating in sexual activity that could lead to pregnancy, all study volunteers must agree that AT LEAST ONE of the following reliable methods of contraception will be used while they are receiving protocol-specified medications and for 6 weeks after stopping the medications:

- Condoms^1^ (male or female) with or without a spermicidal agent
- Diaphragm or cervical cap with spermicide
- IUD

^1^ Condoms are recommended because their appropriate use is the only contraception method effective for preventing HIV transmission.

NOTE: Use of hormonal contraceptives is prohibited during this study because of the potential for drug interactions.

All study volunteers who are not of reproductive potential (e.g., women who have been post-menopausal for at least 24 consecutive months, or women who have undergone surgical sterilization [e.g., hysterectomy and/or bilateral oophorectomy or salpingotomy], or men who have documented azoospermia) are eligible without requiring the use of contraception. Acceptable documentation of sterilization or menopause consists of written or oral documentation communicated by a clinician or a clinician’s staff of one of the following:

- - - - Physician report/letter
      - Operative report or other source documentation in the patient record (a laboratory report of azoospermia is required to document successful vasectomy)
      - Discharge summary
      - Laboratory report of azoospermia
      - FSH measurement elevated into the menopausal range as established by the reporting laboratory.

9. Drug transporter gene *ABCB1* position 3435 genotype either homozygous C/C or homozygous T/T.

10. Self-reported white (of European ancestry), AND both parents of white European ancestry.^a^

^a^ NOTE: Because *ABCB1* T/T genotype is relatively infrequent among individuals of African decent, it is not feasible to perform this study in such individuals because screening would require an extremely large number of individuals. However, if associations are found in this study, future studies may extend to other populations.

## 4.0.3 Exclusion Criteria

1. Within 14 days prior to entry, use of any medication that is known to be a potent inducer (e.g. rifampin) or inhibitor (e.g. ritonavir) of CYP3A or UGT1A1 (refer to manufacturers’ package inserts for individual drugs).

2. Anticipated need to take any medication that is known to be a potent inducer or inhibitor of CYP3A or UGT1A1 during the study.

3. Active drug use or dependence that, in the opinion of the investigator, would interfere with adherence to study requirements.

4. Inability to abstain from alcohol-containing beverages, grapefruit, and grapefruit juice for the duration of the study.

5. Serious illness that, in the opinion of the investigator, would interfere with study participation.

6. Hospitalization for any reason or therapy for serious illness within 14 days prior to study entry.

7. History of hypersensitivity to study drug or its formulation.

8. As determined by the investigator, a significant active or previous history of cardiovascular, renal, liver, hematologic, neurologic, gastrointestinal, psychiatric, endocrine, or immunologic disease(s). This is inclusive of chronic illnesses such as hypertension, coronary artery disease, arthritis, diabetes, any chronic gastrointestinal conditions that may affect drug absorption, etc.

9. Breast-feeding.

10. Evidence of CNS infection or space occupying lesion by history or physical examination.

11. History of significant CNS disorder such as trauma, congenital malformations or genetic disorders.

12. Prisoners or subjects who are compulsorily detained (involuntarily incarcerated) for treatment of either a psychiatric or physical (e.g., infectious disease) illness must not be enrolled into this study.

13. *ABCB1* position 3435 C/T heterozygosity during screening evaluation.

1. **Enrollment/Randomization**

There is no randomization involved in this study. Subjects will be screened and registered for enrollment at the Vanderbilt AIDS Clinical Trials Center (ACTC).

Vanderbilt ACTC

345 24th Ave North, Suite 105

Nashville, TN 37203

Phone: 615-467-0154

FAX: 615-467-0158

Potential study participants will be recruited by word of mouth, by email advertisement through the Vanderbilt system and flyers that will be pre-approved by the Vanderbilt IRB.

ACTC research staff will give a copy of the consent form to potential participants, will discuss study procedures, risks and benefits of the study, and will obtain written informed consent. After signing the informed consent document, subjects will have blood drawn for screening laboratory tests.

1. **Study Procedures** (Refer to Appendix A for Schedule of Events)

**6.0.1 Lumbar puncture**

Subjects will be placed on their side with knees drawn upwards and lumbar spinal space L2-3 or L3-4 will be located; whereupon the back will be cleaned with surgical antiseptic and a local anesthesia will be injected to numb the insertion area.

A 22 gauge spinal needle will be used to obtain lumbar CSF. Approximately 15 mL of CSF will be obtained for RGV assays.

NOTE: See Appendix for CSF sample processing instructions.

**6.0.2. Serial blood collection by indwelling catheter**

An indwelling venous catheter will be placed for pharmacokinetic sampling in a peripheral arm vein.

Blood samples will be collected at intervals through a saline lock (or occasionally by venipuncture if the saline lock does not allow blood to be drawn).

Each blood sample will be promptly placed on wet ice until refrigerated.

For plasma PK, samples will then be centrifuged at 4^O^C at 800xg for 10 minutes within one hour of collection.

Plasma will be harvested from the collection tube and 4 aliquots of each will be transferred into pre-labeled tubes and stored frozen at ‑70^O^C.

For PBMCs, whole blood samples will immediately be placed in a wet ice bath, and processed as described in Appendix C.

**6.0.3 Adherence assessment**

An adequate supply of study medication will be dispensed to last for 7 days (14 pills). All unused pills should be returned to study personnel. At the Day 6 or 7 visit (before PK sampling) study personnel will perform pill counts.

Daily medication logs, completed by each subject, and pill counts will be used for adherence monitoring. On Days 1, 2, or 3 subjects may miss NO MORE THAN ONE DOSE of study drug. On Days 4, 5, and 6 subjects MUST NOT MISS ANY DOSE of study drug.

Any subject who fails to meet these criteria will discontinue study medication. The subject will be taken off study.

**6.0.4 Summary of study events by day**

"Day 1" is the day that study drug is first administered.

Days -365 to -1

1. Obtain written informed consent

2. Venipuncture to obtain blood sample for *ABCB1* genotype.

NOTE: The -365 day window is intended to allow flexibility so that *ABCB1* genotype assays can be batched to increase genotyping efficiency and to accommodate subjects’ schedules. The actual time interval from consenting to Day 1 will be far less than 90 days in most participants.

Days -30 to -1

1. Venipuncture to obtain blood sample for screening safety lab tests, HIV serology.

2. History and physical examination.

3. Neurological examination.

4. Routine screening laboratory assay.

4. Urine pregnancy test (Women only. Must have negative test before taking study medications).

Day 1

1. Urine or Serum pregnancy test (Women only. Must have negative test before beginning study drug).

2. Provide participant with medication diary to document timing of every dose of RGV.

3. Begin RGV 400 mg orally every 12 hours. The first dose is at time specified by study personnel on Day 1.

Days 2-6

1. Continue RGV 400 mg orally every 12 hours in the AM and in the PM, as instructed.

Day 6

1. Arrive at VICTR-CRC in evening, at time specified.

2. Venipuncture to obtain blood sample for safety lab tests.

3. Provide snack 30min. prior to study drug administration.

4. Collect medication diary from participant, and assess adherence.

5. Continue RGV 400 mg orally every 12 hours. The scheduled PM dose of RGV 400 mg should be observed by VICTR-CRC staff and documented.

6. Leave the VICTR-CRC after taking the observed dose of RGV

NOTE: Under rare circumstances the participant may stay in the VICTR-CRC overnight on Day 6.

NOTE: Participant needs to be fasting 8 hours before the final scheduled AM dose of RGV. Only water and coffee with no cream or sugar are allowed.

Day 7

1. Arrive at VICTR-CRC at 6 AM. Admit as inpatient.

2. Day 6 lab results reviewed.

3. Fasting is required for an additional 3 hours after final dose of RVG. Water and coffee with no cream or sugar are allowed. A light regular meal will be given 3 hours after final dose of RVG.

4. Consent form re-signed for LP.

5. Place an indwelling venous catheter in a peripheral arm vein.

6. Serial plasma and PBMC sampling for RGV PK.

NOTE: See Appendix for timing and processing of plasma and PBMC samples.

7. Lumbar puncture 4 hours after the morning RGV dose.

Day 8

1. Plasma and PBMC sample for RGV PK (24 hours ± 1 hour after Day 7 RGV dose).
2. After sample is obtained, discharge participant from the VICTR-CRC.

Day 9

1. Plasma and PBMC sample for RGV PK (48 hours ± 1 hour after Day 7 RGV dose).

Day 14 (± 2 days)

1. Contact participant by telephone (or email if the participant prefers) to make sure there were no subsequent problems related to study participation.

**6.0.5 Study medications**

The following regimen will be used in this study. All doses will be administered with food (except fasting for 8 hours prior to the last dose). There will be no dosage adjustment for weight. Subjects who complete the study will receive study drug for 7 days.

Raltegravir 400 mg orally PO every 12 hours

1. **Risks**

**Potential risks associated with CSF sampling by lumbar puncture**

Lidocaine, used locally during the lumbar puncture initiation, may cause a burning sensation. Local lidocaine injection very rarely enters the bloodstream. There is a risk that lidocaine may cause cardiac arrhythmias. CSF collection may cause bruising at the site, and may result in headache following the procedure. The risks associated with lumbar puncture include most commonly mild transient backache during the procedure, and headache subsequent to the procedure. While these are usually mild to moderate in nature and go away in a day or two, they may last longer (10 days) and have been occasionally more severe (requiring a dextran patch to bring resolution.) Rare and unexpected but potentially more serious side effects from having spinal fluid withdrawn are infection, nerve root damage, and bleeding at the puncture site. The lumbar puncture may produce, on occasion, a sharp, acute, very brief (10-30 seconds) pain in the nerve distribution to the thigh muscle or groin that remits with needle removal. The risks associated with the dextran patch procedure are the same as those associated with the catheterization procedure itself and in addition some individuals will develop transient (several days) post-patch ringing in their ears and a low-grade fever. Headache may also be treated with caffeine sodium benzoate that is associated with increased alertness and jitteriness. There is a negligible risk of infection from having blood samples drawn, and some risk of bruising and discomfort at puncture sites.

Should headache occur, the subject will remain at bed rest until it is gone. Virtually all headaches related to lumbar CSF sampling are posture-related and can be ameliorated by bed rest. Hydration and analgesia will be used as well. If headache occurs before the participant is discharged from the VICTR-CRC, caffeine sodium benzoate (500mg, 2 hour IV drip) will be recommended as a primary treatment. If treatment is successful, the subject will be asked to remain for a minimum of 4 hours rest and observation on the VICTR-CRC. If the first effort at treatment is unsuccessful, 8 hours for rest and observation will be recommended before a second effort at treatment. The second recommended treatment could be a repeated injection of caffeine, or a blood or dextran patch. If headache occurs after discharge from the VICTR-CRC, and is refractory to hydration and analgesics, a blood or dextran patch will be used. The choice of blood versus dextran patch will be based on the clinical judgment of the anesthesiologist performing the procedure. The blood or dextran patch typically brings nearly instantaneous resolution of the headache. All patients will be contacted one week following lumbar puncture to obtain follow-up safety information.

**Potential side effects of study medications**

Raltegravir is very well tolerated, without apparent untoward side effects. However, there may be unknown or unanticipated adverse effects.

1. **Reporting of Adverse Events or Unanticipated Problems involving Risk to Participants or Others**

All serious adverse events or other unanticipated problems involving risk to participants or others will be reported per FDA guidelines and to the Vanderbilt IRB within 48 hours of them being identified. All Grade 3 or higher adverse events will be reported to the Vanderbilt IRB.

A serious adverse event or reaction is any untoward medical occurrence that:

- Results in death;
- Is life-threatening-

(Defined as an event in which the patient or subject was at risk of death at the time of the event; it does not refer to an event which hypothetically might have caused death if it were more severe.);

- Requires inpatient hospitalization or prolongation of existing hospitalization;
- Results in a persistent or significant disability/incapacity;
- Is a congenital anomaly or birth defect;
- Results in the development of drug dependency or drug abuse;
- Is an important medical event-

(Defined as a medical event(s) that may not be immediately life-threatening or result in death or hospitalization but, based upon appropriate medical and scientific judgment, may jeopardize the patient/subject or may require intervention (e.g., medical, surgical) to prevent one of the other serious outcomes listed in the definition above. Examples of such events include, but are not limited to, intensive treatment in an emergency room or at home for allergic bronchospasm; blood dyscrasias or convulsions that do not result in hospitalization.

Adverse events will be graded using the National Institute of Allergy and Infectious Diseases Division of AIDS toxicity scale (http://rcc.tech-res-intl.com/tox_tables.htm). Dr. Haas holds the IND for this study. He will be made promptly aware of any SAEs that occur during the study. In addition, he is responsible for making sure that any SAEs are promptly reported to the FDA by his research staff.

1. **Study Withdrawal/Discontinuation**

Study subjects may be withdrawn from the study for any of the following reasons:

- Drug-related toxicity
- Failure to take study medications as instructed
- Clinical reasons believed life threatening by the physician
- Request by the subject to withdraw
- Request of the primary care provider if s/he thinks the study is no longer in the best interest of the subject
- Subject judged by the investigator to be at significant risk of failing to comply with the provisions of the protocol as to cause harm to self or seriously interfere with the validity of the study results
- Inability to obtain CSF for technical reasons
- Subjects who become prisoners or become involuntarily incarcerated for the treatment of either a psychiatric or physical (e.g., infectious disease) illness within the first 14 days of study participation.

1. **Statistical Considerations**

**Pharmacokinetic parameters**

The concentration of RGV in CSF and plasma will be determined by GC/MS assay. Plasma pharmacokinetic parameter estimates will be determined using a non-compartmental approach (WinNonlin version 4.01, Pharsight Corp., Mountain View, CA.). Calculated pharmacokinetic parameters will be: area-under-the-curve (AUC_0-12h_), maximum plasma concentration (C_max_), minimum plasma concentration (C_min_), time to C_max_ (T_max_), oral clearance (CL/F), terminal apparent distribution volume (V_z_/F), and elimination half-life (t_1/2_). AUC_0-12h_ will be determined using the linear/log trapezoidal method. C_max_, T_max_, and C_12h_ will be taken directly from the observed concentration-time data. CL/F will be calculated as dose/AUC_0-12h_. V_z_/F will be calculated as dose divided by the product of the elimination rate constant (λ_z_) and AUC_0-12h_. The elimination rate constant will be determined by linear regression of the terminal elimination phase concentration-time points; t_1/2_ will be calculated as ln(2)/λ_z_.

**Statistical analyses**

There are no data to reliably predict for RGV the ratio of CSF C_4h_ to plasma AUC_0-12h_ values. We expect this ratio to be quite low. Our power calculation is therefore only a rough estimate. If we assume that PK values will be normally distributed within each *ABCB1* genotype group, a standard deviation of 0.001 for the CSF-to-plasma ratio, and using Student’s t test, studying 17 individuals in each genotype group will allow us to detect with 80% power and an alpha of 0.05 a difference of 0.001 between *ABCB1* C/C and T/T genotype groups. Because we anticipate that not all participants will be evaluable, we will enroll 3 additional individuals in each of the genotype groups, for a total of 20 per genotype group.

The primary outcomes of interest will be ratios of the CSF RGV C_4h_ value to the plasma RGV C_max_, C_min_ and AUC_0-12h_ values. If PK parameters are normally distributed within each genotype group, then genotype groups will be compared using Student’s t test. If PK parameters are not normally distributed within each genotype group, then genotype groups will be compared using nonparametric tests such as the Mann-Whitney U test.

1. **Privacy/Confidentiality Issues**

All laboratory specimens, evaluation forms, reports, and other records that leave the site will be identified by coded number only to maintain subject confidentiality. All records will be kept locked. All computer entry and networking programs will be done with coded numbers only.

1. **Follow-up and Record Retention**

Study records will be retained for at least 10 years.

**Appendix A:** ^Schedule of events^

|  |  |  |  |  |  | Day |  |  |  |  |  |  |
| --- | --- | --- | --- | --- | --- | --- | --- | --- | --- | --- | --- | --- |
|  | Screen -365 to -1 | Screen -30 to -1 | 1 | 2 | 3 | 4 | 5 | 6 | 7 | 8 | 9 | 14 (±2) |
| **All subjects** |  |  |  |  |  |  |  |  |  |  |  |  |
| Informed consent | X |  |  |  |  |  |  |  | X |  |  |  |
| *ABCB1* genotype | X |  |  |  |  |  |  |  |  |  |  |  |
| Routine safety labs (creatinine, CBC with diff, platelet count, ALT, AST, alkaline phosphatase, bilirubin, creatinine kinase (CK)) |  | X |  |  |  |  |  | X |  |  |  |  |
| HIV serology |  | X |  |  |  |  |  |  |  |  |  |  |
| Urine or Serum pregnancy test (women only) |  | X | X |  |  |  |  |  |  |  |  |  |
| Take raltegravir 400 mg every 12 hours |  |  | X | X | X | X | X | X | X |  |  |  |
| VICTR-CRC visit (admission to VICTR-CRC in rare circumstances) |  |  |  |  |  |  |  | X | X |  |  |  |
| CSF for total protein, cell count |  |  |  |  |  |  |  |  | X |  |  |  |
| Phone/or email follow-up |  |  |  |  |  |  |  |  |  |  |  | X |
| **Clinical evaluations** |  |  |  |  |  |  |  |  |  |  |  |  |
| Complete physical exam (includes height, weight, funduscopic, neurologic exam) |  | X |  |  |  |  |  |  |  |  |  |  |
| Funduscopic, targeted neurologic exam before LP. |  |  |  |  |  |  |  |  | X |  |  |  |
| Medication adherence assessment; distribution/collection of medication log |  |  | X |  |  |  |  |  | X |  |  |  |
| **Pharmacokinetic (PK) sampling** |  |  |  |  |  |  |  |  |  |  |  |  |
| Serial plasma sampling for PK |  |  |  |  |  |  |  |  | X | X | X |  |
| PBMC sampling for PK |  |  |  |  |  |  |  |  | X | X | X |  |
| Lumbar puncture for PK 4 hours after morning RGV dose |  |  |  |  |  |  |  |  | X |  |  |  |

**Appendix B:** References

Reference List

(1) Lee CG, Gottesman MM. HIV-1 protease inhibitors and the MDR1 multidrug transporter. Journal of Clinical Investigation 1998;101:287-8.

(2) Srinivas RV, Middlemas D, Flynn P, Fridland A. Human immunodeficiency virus protease inhibitors serve as substrates for multidrug transporter proteins MDR1 and MRP1 but retain antiviral efficacy in cell lines expressing these transporters. Antimicrobial Agents & Chemotherapy 1998;42:3157-62.

(3) Kim RB, Fromm MF, Wandel C, et al. The drug transporter P-glycoprotein limits oral absorption and brain entry of HIV-1 protease inhibitors. Journal of Clinical Investigation 1998;101:289-94.

(4) Kim AE, Dintaman JM, Waddell DS, Silverman JA. Saquinavir, an HIV protease inhibitor, is transported by P-glycoprotein. Journal of Pharmacology & Experimental Therapeutics 1998;286:1439-45.

(5) Polli JW, Jarrett JL, Studenberg SD, et al. Role of P-glycoprotein on the CNS disposition of amprenavir (141W94), an HIV protease inhibitor. Pharmaceutical Research16:1206-12.

(6) Washington CB, Wiltshire HR, Man M, et al. The disposition of saquinavir in normal and P-glycoprotein deficient mice, rats, and in cultured cells. Drug Metabolism & Disposition 2000;28:1058-62.

(7) Letendre S, et al. Better Antiretroviral Penetration into the Central Nervous System Is Associated with Lower CSF Viral Load . Presented at 13th Conference on Retroviruses and Opportunistic Infections, February 2006, Denver, CO Abstract 74 2006.

(8) Marzolini C, Paus E, Buclin T, Kim RB. Polymorphisms in human MDR1 (P-glycoprotein): recent advances and clinical relevance. Clin Pharmacol Ther 2004;75:13-33.

(9) Kimchi-Sarfaty C, Oh JM, Kim IW, et al. A "silent" polymorphism in the MDR1 gene changes substrate specificity. Science 2007;315:525-8.

(10) Haas DW, Smeaton LM, Shafer RW, et al. Pharmacogenetics of Long-Term Responses to Antiretroviral Regimens Containing Efavirenz and/or Nelfinavir: An Adult AIDS Clinical Trials Group Study. J Infect Dis 2005;192:1931-42.

(11) Fellay J, Marzolini C, Meaden ER, et al. Response to antiretroviral treatment in HIV-1-infected individuals with allelic variants of the multidrug resistance transporter 1: a pharmacogenetics study. Lancet 2002;359:30-6.

(12) Motsinger AA, Ritchie MD, Shafer RW, et al. Multilocus genetic interactions and response to efavirenz-containing regimens: an Adult AIDS Clinical Trials Group study. Pharmacogenet Genomics 2006;16:837-45.

(13) Ritchie MD, Haas DW, Motsinger AA, et al. Drug transporter and metabolizing enzyme gene variants and nonnucleoside reverse-transcriptase inhibitor hepatotoxicity. Clin Infect Dis 2006;43:779-82.

(14) Haas DW, Bartlett JA, Andersen JW, et al. Pharmacogenetics of nevirapine-associated hepatotoxicity: an Adult AIDS Clinical Trials Group collaboration. Clin Infect Dis 2006;43:783-6.

(15) Haas DW, Ribaudo HJ, Kim RB, et al. Pharmacogenetics of efavirenz and central nervous system side effects: an Adult AIDS Clinical Trials Group study. AIDS 2004;18:2391-400.

(16) Ribaudo HJ, Haas DW, Tierney C, et al. Pharmacogenetics of plasma efavirenz exposure after treatment discontinuation: an Adult AIDS Clinical Trials Group Study. Clin Infect Dis 2006;42:401-7.

(17) Hulgan T, Haas DW, Haines JL, et al. Mitochondrial haplogroups and peripheral neuropathy during antiretroviral therapy: an adult AIDS clinical trials group study. AIDS 2005;19:1341-9.

(18) Hulgan T, Haas D, Haines J, et al. Non-synonymous Mitochondrial DNA Polymorphisms and Peripheral Neuropathy during NRTI Therapy in ACTG Study 384. Presented at 13th Conference on Retroviruses and Opportunistic Infections, February 2006, Denver, CO, abstract 350 2006.

(19) Kallianpur AR, Hulgan T, Canter JA, et al. Hemochromatosis (HFE) gene mutations and peripheral neuropathy during antiretroviral therapy. AIDS 2006;20:1503-13.

(20) Haas DW, Geraghty DE, Andersen J, et al. Immunogenetics of CD4 Lymphocyte Count Recovery during Antiretroviral Therapy: An AIDS Clinical Trials Group Study. J Infect Dis 2006;194:1098-107.

(21) Haas DW, Stone J, Clough LA, et al. Steady-state pharmacokinetics of indinavir in cerebrospinal fluid and plasma among adults with human immunodeficiency virus type 1 infection. Clinical Pharmacology & Therapeutics 2000;68:367-74.

(22) Haas DW, Clough LA, Johnson BW, et al. Evidence of a source of HIV type 1 within the central nervous system by ultraintensive sampling of cerebrospinal fluid and plasma. AIDS Research & Human Retroviruses 2000;16:1491-502.

(23) Haas DW, Weller S, Johnson B, et al. CSF Penetration of Acyclovir (ACV) and its Metabolites after High Dose Valacyclovir (VACV) Administration. Presented at 43rd Interscience Conference on Antimicrobial Agents and Chemotherapy, Chicago, IL 2003.

(24) Haas DW, Johnson BW, Spearman P, et al. Two phases of HIV RNA decay in cerebrospinal fluid during initial days of multidrug therapy. Neurology 2003;61:1391-6.

(25) Haas DW, Johnson B, Nicotera J, et al. Effects of ritonavir on indinavir pharmacokinetics in cerebrospinal fluid and plasma. Antimicrob Agents Chemother 2003;47:2131-7.

(26) Harrington PR, Haas DW, Ritola K, Swanstrom R. Compartmentalized human immunodeficiency virus type 1 present in cerebrospinal fluid is produced by short-lived cells. J Virol 2005;79:7959-66.

# Appendix C: Specimen Collection and Processing

| **Assay/Procedure** | Tube Type or Specimen Type and Quantity | Derivative | Processing Instructions | Aliquots/Storage | Shipping Address, Frequency, and Other Instructions |
| --- | --- | --- | --- | --- | --- |
| Plasma raltegravir assays^a^ | Whole blood in 10 mL K3 EDTA tube: 1 tube per scheduled draw time. | Plasma | Place sample on ice promptly after collection. Within 1 hour spin blood at 800xg for 10 minutes at 4^O^C; remove plasma and freeze 4 equal aliquots (at least 1 mL each) at ‑70°C. | 4 equal aliquots (at least 1 mL plasma per cryovial). Store immediately at ‑70°C. | One aliquot from each timepoint will be batch shipped to:  Edward P. Acosta, Pharm.D.  Antiviral Laboratory Director  Lab Contact:  Kedria Walker, B.S.  Division of Clinical Pharmacology  University of Alabama at Birmingham  Volker Hall Room 101A  1670 University Boulevard  Birmingham, AL 35294-0019  Phone: 205-975-2461  Fax: 205-934-6201  Include ample dry ice to ensure frozen delivery of samples on next business day. Notify laboratory when shipment is placed.  The remaining aliquots will continue to be stored at -70^O^C. |
| **CSF raltegravir assays**^a^ | 15 mL Cerebrospinal fluid collected on ice. | CSF | Place CSF tube promptly on ice. Pipette at least 5 mL UNSPUN CSF (at least 1 ml per tube) directly into at least 5 separate 2-mL Sarstedt Micro tubes (cryovials, catalogue no. 72.694.006). Label tubes “UNSPUN”  Spin the remaining CSF (at least 6 mL) at 800x*g* for 10 minutes at 4^O^C in a 15 mL conical tube. Pipette supernatant into at least 5 separate cryovials (at least 1 mL per tube). Label tubes “SPUN”. Take care not to disturb the pellet (even if no pellet is visible) | 10 equal aliquots of CSF (at least 5 unspun and 5 spun). Store immediately at ‑70°C. | Two SPUN aliquots from each timepoint will be batch shipped to:  Edward P. Acosta, Pharm.D.  Antiviral Laboratory Director  Lab Contact:  Kedria Walker, B.S.  Division of Clinical Pharmacology  University of Alabama at Birmingham  Volker Hall Room 101A  1670 University Boulevard  Birmingham, AL 35294-0019  Phone: 205-975-2461  Fax: 205-934-6201 |
| ***ABCB1* genotype^b^** | Whole blood in 10 ml K3 EDTA tube |  | Collect whole blood into Sarstedt K3 EDTA Monovette. Either store within 12 hours at ‑70°C, or deliver ambient to Vanderbilt DNA Resources Core.  Description:  Sarstedt K3 EDTA Monovette  Product Number: 01.1605.100  Telephone Number: 1-700-257-5101 | N/A | Vanderbilt DNA Resources Core  Attn: Cara Sutcliffe  518 Light Hall  Vanderbilt University, Medical Center  Nashville, TN 37232-0700  Telephone: 615-936-2744  Fax: 615-936-3817  E-mail: core@phg.mc.vanderbilt.edu |
| **CBC with diff and platelets** | Whole blood in 4 mL purple-top EDTA tube |  | Send in real-time to clinical lab for assay |  | LabCorp |
| **Creatinine, ALT, AST, alkaline phosphatase, bilirubin, Creatinine Kinase (CK)** | Whole blood in 8.5ml red marble-top tube |  | Send in real-time to clinical lab for assay |  | LabCorp |
| **HIV Elisa** | 8.5ml red marble-top tube |  | Send in real-time to clinical lab for assay |  | LabCorp |
| **CSF total protein , cell count** | Unspun CSF, 2 mL. |  | Send in real-time to clinical lab for assay |  | Vanderbilt Univ Med Ctr  Diagnostic Lab  4605 TVC (5310)  Deliver to lab immediately  Cell count test code (CSF) CPT code 89051  Protein test code (SFP) CPT code 84157 |
| **PBMC raltegravir assays**^a^ | Whole blood in 10 mL K3 EDTA tube: 2 tubes per scheduled draw time. |  | Whole blood for PBMCs will be promptly placed in an ice bath. PBMCs will be isolated within 1 hour at 4^O^C and counted with a hemacytometer. Cell density will be adjusted to a concentration of 2 x 10^6^ cells/ml with ice-cold phosphate buffered saline, then aliquotted into as many 1 mL aliquots as possible, and promptly placed at -80^O^C. Discard the final aliquot if it has less than 2 x 10^6^ cells/ml. |  | Two PBMC aliquots from each timepoint will be batch shipped to:  Edward P. Acosta, Pharm.D.  Antiviral Laboratory Director  Lab Contact:  Kedria Walker, B.S.  Division of Clinical Pharmacology  University of Alabama at Birmingham  Volker Hall Room 101A  1670 University Boulevard  Birmingham, AL 35294-0019  Phone: 205-975-2461  Fax: 205-934-6201 |

^a^ Raltegravir in CSF and plasma will be assayed by GC/MS assay.

^b^ Genomic DNA will be extracted from whole blood using PUREGENE (Gentra Systems, Inc., Minneapolis, MN). Genotyping will be accomplished with the ABI PRISM 7900 HT Sequence Detection System (Applied Biosystems, Inc., Foster City, CA), using customized TaqMan^TM^ assays.

**Appendix D:** Upper limit weight cut-off for study participation (body mass index = 30)

| **Feet** | | **Inches** | | **Weight cutoff (pounds)** | |
| --- | --- | --- | --- | --- | --- |
| 4 | ft | 0 | in | 98.3 | lbs |
|  | ft | 1 | in | 102.5 | lbs |
|  | ft | 2 | in | 106.7 | lbs |
|  | ft | 3 | in | 111.0 | lbs |
|  | ft | 4 | in | 115.4 | lbs |
|  | ft | 5 | in | 119.9 | lbs |
|  | ft | 6 | in | 124.4 | lbs |
|  | ft | 7 | in | 129.1 | lbs |
|  | ft | 8 | in | 133.8 | lbs |
|  | ft | 9 | in | 138.6 | lbs |
|  | ft | 10 | in | 143.6 | lbs |
|  | ft | 11 | in | 148.5 | lbs |
| 5 | ft | 0 | in | 153.6 | lbs |
|  | ft | 1 | in | 158.8 | lbs |
|  | ft | 2 | in | 164.0 | lbs |
|  | ft | 3 | in | 169.4 | lbs |
|  | ft | 4 | in | 174.8 | lbs |
|  | ft | 5 | in | 180.3 | lbs |
|  | ft | 6 | in | 185.9 | lbs |
|  | ft | 7 | in | 191.6 | lbs |
|  | ft | 8 | in | 197.3 | lbs |
|  | ft | 9 | in | 203.2 | lbs |
|  | ft | 10 | in | 209.1 | lbs |
|  | ft | 11 | in | 215.1 | lbs |
| 6 | ft | 0 | in | 221.2 | lbs |
|  | ft | 1 | in | 227.4 | lbs |
|  | ft | 2 | in | 233.7 | lbs |
|  | ft | 3 | in | 240.0 | lbs |
|  | ft | 4 | in | 246.5 | lbs |
|  | ft | 5 | in | 253.0 | lbs |
|  | ft | 6 | in | 259.6 | lbs |
|  | ft | 7 | in | 266.3 | lbs |
|  | ft | 8 | in | 273.1 | lbs |
|  | ft | 9 | in | 280.0 | lbs |
|  | ft | 10 | in | 286.9 | lbs |
|  | ft | 11 | in | 294.0 | lbs |
| 7 | ft | 0 | in | 301.1 | lbs |
|  | ft | 1 | in | 308.3 | lbs |

**On-line BMI calculator available at** [**http://www.nhlbisupport.com/bmi/**](http://www.nhlbisupport.com/bmi/)

# Appendix E: Pharmacokinetic (PK) Evaluations

Subjects will arrive at the VICTR- Clinical Research Center (VICTR-CRC) before 6 PM the evening prior to the PK sampling visit. Study drugs will be given at the same time as the subject’s usual dose. Subjects should take their scheduled doses of study drugs AT THE SAME TIMES for at least 2 days prior to the PK sampling visit. This should ideally be at 7 AM and 7 PM. The subject should then take the RGV at the same time (7 AM) and the second dose 12 hours later (7 PM).

After the participant takes the observed dose of RGV on the evening of day 6, the participant will leave VICTR-CRC to return at 6 AM the next morning for a 24-hour VICTR-CRC stay. Under rare circumstances the participant may stay in VICTR-CRC overnight on Day 6.

Before initiating the evaluations listed below, study personnel should review the daily medication log with the subject and conduct a pill count. If the subject meets adherence requirements, the plasma and PBMC PK evaluations can be conducted.

If the subject does not meet adherence requirements, no sample should be drawn. If adherence requirements are not met, study drug should be discontinued and the subject should be taken off study after completion of the safety follow-up visit.

All plasma and PBMC PK samples must be drawn ideally at the times listed below. The actual collection time must be recorded on the case report form.

*PK Sampling on Days 6 and 7 in GCRC:*

| Hour | Activity | Specimen Drawn |
| --- | --- | --- |
| **DAY 6** |  |  |
| 6:00 PM | Arrive at VICTR-CRC. Venipuncture for safety labs. |  |
| 6:30 | Snack |  |
| 7:00 PM | Administer raltegravir 400 mg PO. Record administration time. Collect medication diary. |  |
| 7:00 PM | Leave VICTR-CRC |  |
| **DAY 7** |  |  |
| -1 (6:00 AM) | Arrive at VICTR-CRC |  |
| -1 | Vital Signs |  |
| -0.5 (6:30 AM) | Indwelling venous catheter insertion |  |
| -0.25 (7:45 AM) | Baseline plasma PK sample | 10 mL EDTA |
| 0 (7:00 AM) | Administer raltegravir 400 mg PO **EXACTLY 12 HOURS AFTER PREVIOUS DOSE**; start clock; record administration time; document hour and day of last food. |  |
| 1 | plasma PK sample | 10 mL EDTA |
| 2 | plasma PK sample | 10 mL EDTA |
| 3 | plasma PK sample | 10 mL EDTA |
| 3 | Light regular meal |  |
| 4 | Lumbar puncture for CSF | 15 mL CSF |
| 4 | plasma PK sample | 10 ml EDTA |
| 4 | PBMC PK sample | 20 mL EDTA |
| 5 | plasma PK sample | 10 mL EDTA |
| 6 | plasma PK sample | 10 mL EDTA |
| 8 | plasma PK sample | 10 mL EDTA |
| 8 | Regular diet ad lib hereafter |  |
| 12 | plasma PK sample | 10 mL EDTA |
| 16 | plasma PK sample | 10 mL EDTA |
| **DAY 8** |  |  |
| 20 (3:00 AM) | plasma PK sample | 10 mL EDTA |
| 24 (7:00 AM) | plasma PK sample | 10 mL EDTA |
| 24 | PBMC PK sample | 20 mL EDTA |
| 24.25 | Remove venous catheter |  |
| 24.25 | Discharge subject from VICTR-CRC |  |
| **DAY 9** |  |  |
| 48 (7:00 AM) | plasma PK sample | 10 mL EDTA |
| 48 | PBMC PK sample | 20 mL EDTA |

IN-PATIENT MENU

A light meal of subjects’ choice will be provided 3 hours after the study drug is given.

NOTE: Subjects are NOT to be given grapefruit or grapefruit juice.

APPENDIX V

INFORMED CONSENT

**Genetic Predictors of Raltegravir Penetration into Cerebrospinal Fluid**

SHORT TITLE FOR THE STUDY: Raltegravir Penetration into CSF

INTRODUCTION

You are being asked to take part in the research study named above. The study will include only adults who do not have HIV (the virus that causes AIDS). To be in this study you must also be of white European ancestry, and both your parents must be of white European ancestry.

This study is sponsored by Merck and Company, Inc. The doctor in charge of this study at Vanderbilt is David W. Haas, MD. Before you decide if you want to be a part of this study, we want you to know about the study.

This is a consent form. It gives you information about this study. The study staff will talk with you about this information. You are free to ask questions about this study at any time. If you agree to take part in this study, you will be asked to sign this consent form. You will get a copy to keep.

WHY IS THIS STUDY BEING DONE?

The purposes of this study are:

1. To find out how much of the drug raltegravir (RGV) gets into cerebrospinal fluid (CSF, the fluid that surrounds your brain and spinal cord) compared to how much gets into the blood, and to find out if a normal variation in a certain gene called *ABCB1* affects how much gets into the CSF. This will be done by measuring RGV in your blood at different times after you take the drug, and one time in your CSF.

Raltegravir is a drug that is approved by the Food and Drug Administration (FDA) to treat HIV infection. The dose you will take during the study is the same dose that is used to treat HIV infection. The study will involve taking RGV 400 mg every 12 hours for 7 days.

WHAT DO I HAVE TO DO IF I AM IN THIS STUDY?

You will take RGV twice-a-day for 7 days. On the evening of the 6^th^ day of RGV you will be asked to go to VICTR- Clinical Research Center (VICTR-CRC). You may choose to stay in the VICTR-CRC overnight, or you may leave the VICTR-CRC and return early the next morning. You will stay in the VICTR-CRC overnight on the evening of the 7^th^ day. Your total time in the VICTR-CRC will be about 24 hours. You will have another study visit on the 9^th^ day. You will also be called for a follow-up contact about 1 week after you stop taking the RGV. A total of about 18 tablespoons (260 mL) of blood will be drawn during the study. The procedures for the study visits are described below.

Screening

If you decide to enroll in this study, you will be asked to sign this consent form. You will come to the clinic to have a screening visit. Eligibility tests (to see whether you are able to take part in the study) will be performed. The screening visit will take about 1 hour.

At this visit you will be asked about your medical history and any medications you have taken in the past. You will have your overall health checked to see if it is safe for you to be in the study. You will have a complete physical exam, not including breast, genital, or rectal exam. You will have about 2 tablespoons of blood drawn from a vein in your arm. Some of your blood will be used for a test of your DNA (genetic test) for a normal variation in a gene called *ABCB1*. The result of this DNA test will be used to decide if you can be in the study. If the DNA test shows that you can be in the study, your blood will also be tested to make sure you are HIV negative. If your HIV test result is positive, you will receive appropriate counseling, and you will not be enrolled in the study. If you want the results of your other blood tests, please ask the study staff and they will provide these to you.

Screening will require 2 visits. During the first visit volunteers will be asked to sign the consent form, and have about 1 teaspoon (7 milliliters) of blood drawn. The second visit may be as much as 12 months later, but probably much sooner. The research staff will tell you whether you will have 1 or 2 screening visits, and when these visits will be.

If you are a woman able to become pregnant, you will have a pregnancy test. Some of the blood drawn for the tests listed above may be used, or you may have a urine sample collected for the pregnancy test. If you are pregnant, you will not be enrolled in the study.

The research staff will explain the procedures involved in the study, including dietary requirements and telephone contacts during the study. You must also agree not to drink alcohol, eat grapefruit, or drink grapefruit juice during the study.

Entry (Day 1)

If you qualify for the study based on results from the screening visit, you will be asked to return to the clinic for an entry visit. At this visit you may have a limited physical exam, depending on whether you have any symptoms. You will be asked about your medical history. If you want to know the results of your safety lab tests please ask the study staff.

If you are a woman able to become pregnant, and you have not had a pregnancy test during the 7 days before you entered the study, you will have a urine or serum pregnancy test on the day of entry. If you are pregnant, you will stop participating in the study.

You will be given a card (Medication Use Log) to write down every day and time you take the study drugs.

You will be given a bottle of the study drug, raltegravir (RGV). At the specified PM time on the night of Day 1, you will take your first dose of RGV.

Study Medication (Days 1-7)

You will take one 400-mg RGV pill every 12 hours for 7 days. On Days 2-6 you will take a dose at in the AM and in the PM as directed.

You will use the Medication Use Log to write down every dose and time you take the study drug.

GCRC Visit (Day 6)

You will come to the VICTR-CRC on the evening of Day 6. You should arrive at the time specified by study personnel. You will return your Medication Use Log to the VICTR-CRC staff. You must also bring your RGV pills to the GCRC.

About 1 teaspoon of blood will be drawn for safety lab tests.

The VICTR-CRC staff will give you a snack to eat, and will then watch you take the scheduled PM dose of RGV from the pills you brought with you.

After taking the RGV you leave the VICTR-CRC and return at 6 AM the next morning.

Blood and CSF Sampling in VICTR-CRC (Day 7)

When you arrive in the VICTR-CRC on Day 7, you will be asked to re-sign the consent form to indicate your willingness to continue in the study and have the following exams and procedures done.

You will have a limited physical exam, depending on whether you have any symptoms.

For the multiple blood samples required, an indwelling catheter (a small, thin tube) will be put into an arm vein on the morning of Day 7 and left in place during your stay in the VICTR-CRC. This will allow the blood samples to be drawn without any additional needle sticks. For each blood draw about 2 teaspoons (10 milliliters) of blood will be taken. This will be done about 12 times. You will have blood drawn to check the level of RGV. About 15 minutes prior to last dose of RGV, a baseline

-0.25 blood sample will be taken and then at 1, 2, 3, 4, 5, 6, 8, 12, 16, 20 and 24 hours after you take RGV to check its levels in your blood. You will have a total of about 12 tablespoons (180 milliliters) of blood drawn during this period. You will leave the VICTR-CRC on the morning of Day 8 after the 24 hour blood draw.

You will be given a light meal by the clinic staff 3 hours after you take last RGV dose on Day 7. About 4 hours after you take RGV on Day 7 in the VICTR-CRC a lumbar puncture (also called spinal tap) will be done to see how much RGV is in your spinal fluid. To do the spinal tap, you will lie on your side, the skin of your lower back will be cleaned and a local painkiller will be given with a needle to briefly numb the area. A long, thin needle will enter the skin in your lower back, passing through muscles, ligaments and the dura (a tough membrane around the spinal cord and spinal fluid sac) to enter the spinal fluid space. About 1 tablespoon of spinal fluid will be removed through the needle. Spinal fluid is the watery liquid around the brain and spinal cord. The human body makes about 500 milliliters of spinal fluid each day. The needle will then be taken out. You should lie on your back at least another hour after the needle is taken out.

If you get a headache after the spinal tap, the doctor may give you painkillers to be used for the first 24 hours. The doctor may give other drugs if needed or thinks that you would be best treated with a “blood patch” or “dextran patch". If this is advised, it will be described again at that time in detail. The doctor doing the procedure will decide whether to use a blood patch or a dextran patch. A blood patch involves drawing a small amount of blood from a vein in your arm, and placing this into your back at the site where the lumbar puncture was done. A dextran patch involves placing a small amount of a strong sugar solution into your back at the site where the lumbar puncture was done. The blood patch or dextran patch is placed into your back using a needle that is almost the same as the one used for doing the lumbar puncture. This time, it is not pressed through the tough membrane called the dura. Instead, the blood patch or dextran patch is placed over the dura. The needle is removed right after the injection. This forms a seal over the leak and usually stops the headache. If a blood patch or dextran patch is needed, this will be done in the VICTR-CRC.

After the lumbar puncture, limits will be placed on your activity, such as no heavy lifting or strong workouts, and will be strongly advised for 24 hours after the collection.

After the morning dose of RGV on Day 7 you will stop taking your study medication.

On the morning of Day 9 you will return to the clinic to have another blood sample drawn. You will have a total of about 2 tablespoons (30 milliliters) of blood drawn at this time.

Follow-Up Contact

About 1 week after you finish taking the study drug the research staff will contact you to make sure you have not had any problems since finishing the study.

Premature Discontinuation

If you stop taking the study drugs before the end of the study, you will be asked to return to the clinic so that the research staff can gather more information about the effects of the drugs, which may be useful for future study subjects. You will have a brief physical exam performed and you will be asked questions about your health since the last study visit.

If you have side effects during the study you may need to return to the clinic for extra visits until the side effects go away.

Other Information

Some of your blood will be stored (labeled only with coded identifiers to protect your privacy) and used for testing that is required for this study.

Some of your blood that is left over after all required study testing is done may be stored (labeled only with coded identifiers to protect your privacy) and used for other research.

HOW MANY PEOPLE WILL TAKE PART IN THIS STUDY?

40 people will take part in this study.

HOW LONG WILL I BE IN THIS STUDY?

You may be in this study for as many as 12 months, but probably much less (about 3 months).

WHY WOULD THE DOCTOR TAKE ME OFF THIS STUDY EARLY?

The study doctor may need to take you off the study early without your permission if:

- the study is cancelled by the U.S. Food and Drug Administration (FDA), the drug company supporting this study, or the site’s Institutional Review Board (IRB). (An IRB is a committee that watches over the safety and rights of research subjects.)
- there are too many side effects in other people in the study.
- the investigator decides that continuing in the study would be harmful to you

The study doctor may also need to take you off the study drug(s) without your permission if:

- you become pregnant
- continuing the study drug(s) may be harmful to you or you have unwanted side effects from the study drugs
- need a treatment that you may not take while on the study
- you are not able to attend the study visits, take the study drug(s), or follow study instructions as required by the study
- you are imprisoned or confined for treatment of an illness.

WHAT ARE THE RISKS OF THE STUDY?

Raltegravir has so far been very safe and well tolerated in studies in healthy volunteers and in people with HIV infection. There have not been any major or life-threatening side effects with this drug. However, this is a new drug and there may be side effects that we do not know about yet. If you have questions concerning study drug side effects, please ask the research staff.

Some side effects you may have include the following:

- upset stomach, vomiting, and diarrhea
- headache
- skin rash

Risk of Blood Draws and Having an Indwelling Catheter

Taking blood may cause some discomfort, bleeding, bruising, and/or swelling where the needle enters the body, and in rare cases, it may result in fainting. It may also make you feel dizzy or lightheaded. There is a small risk of infection or a blood clot from the indwelling catheter.

Risks of Spinal Fluid Sampling

The lumbar puncture may cause back or neck soreness, irritation of nerves coming from the spinal cord, leg pain, infection, such as meningitis, nerve root damage, and bruising in the lower back. Death may result if a person has increased pressure around their brain. A careful physical exam will be used to rule this out. Headache is the most common problem from lumbar puncture. In general, the amount of headache related to the procedure is of fair severity but could be the worst headache you have ever had. The headache usually goes away while you lie flat. While the headache often goes away in a day or two, it can last longer. The doctors expect that about 1 in 10 subjects will get a headache that will respond to drugs. Less than 1 in 20 subjects will have a severe headache that requires a blood patch or dextran patch.

Risk of Lidocaine;

Lidocaine, a numbing drug, may burn or cause a rash, redness or soreness where you get the shot. There is a risk that this drug may cause problems with heart rhythm.

Risks of Potential Treatments for Pain (Blood Patch, Dextran Patch and Drugs)

Should you have a severe or prolonged headache or backache, we will provide proper medical care to help relieve the symptoms. The doctors may prescribe pain pills. If your headache does not respond to drugs, the doctors may perform a blood patch or dextran patch. Risks of the blood patch or dextran patch include neck soreness, irritation of nerves coming from the spinal cord, leg pain, and bruising in the lower back, and infection of the lower back. There is a small risk of infection which could require longer treatment with antibiotics. Other side effects of the blood patch or dextran patch include short-term (a few days) ringing in the ears and a low-grade fever.

Additional Information

There is the risk of serious and/or life-threatening side effects when non-study medications are taken with study drugs. For your safety, you must tell the study doctor or nurse about all the medications you are taking before you start the study and before taking any non-study medications while you are on the study, except for the following. If during the study you have a headache you may take over-the-counter Tylenol, and if you have diarrhea you may take over-the-counter Imodium without first telling the study doctor or nurse.

If you have side effects from any of your study drugs, you may be asked to return to the clinic for additional study visits that may require blood to be drawn for laboratory tests.

ARE THERE RISKS RELATED TO PREGNANCY?

It is not known if the drug or drug combinations in this study harm unborn babies. If you are having sex that could lead to pregnancy, you must agree not to become pregnant or make a woman pregnant.

You and your partner must use a reliable birth control method that you discuss with the study staff. You must continue to use birth control until 6 weeks after stopping the study drugs.

If you can become pregnant, you must have a pregnancy test before you enter this study. The test must show that you are not pregnant. If you think you may be pregnant at any time during the study, tell your study staff right away. The study staff will talk to you about your choices.

Breast-Feeding

It is unknown whether the study drug or study drug combinations pass through the breast-milk and may cause harm to an infant. Women who are breast-feeding may not be in the study.

ARE THERE BENEFITS TO TAKING PART IN THIS STUDY?

This study will be of no direct benefit to you. However, knowledge gained from this study may in the future help people who suffer from AIDS.

WHAT OTHER CHOICES DO I HAVE BESIDES THIS STUDY?

You have the choice of not participating in this study.

WHAT ABOUT CONFIDENTIALITY?

We will do everything we can to protect your privacy. Any publication of this study will not use your name or identify you personally.

People who may review your records include: the U.S. Food and Drug Administration (FDA), the Vanderbilt IRB, the Office for Human Research Protections (OHRP), National Institutes of Health (NIH), study staff, study monitors, the drug company supporting this study, and its designees.

If the study staff learns of possible child abuse and/or neglect or a risk of harm to yourself or others, we will be required to tell the proper authorities.

WHAT ARE THE COSTS TO ME?

There is no cost to you for the study drugs or for study-related clinic visits, examinations, or laboratory tests. Medical costs for treatment outside of this study will be charged to you or your health insurance company.

Taking part in this study may lead to added costs to you and your insurance company. In some cases it is possible that your insurance company will not pay for these costs because you are taking part in a research study.

WILL I RECEIVE ANY PAYMENT?

You will be paid $600 after you complete the study. You will be paid less if you do not complete all the study visits.

WHAT HAPPENS IF I AM INJURED?

If you are injured as a result of being in this study, you will be given immediate treatment for your injuries. The cost for this treatment will be charged to you or your insurance company. There is no program for compensation through this institution. You will not be giving up any of your legal rights by signing this consent form.

WHAT ARE MY RIGHTS AS A RESEARCH SUBJECT?

Taking part in this study is completely voluntary. You may choose not to take part in this study or you may leave this study at any time. You will be treated the same no matter what you decide.

We will tell you about new information from this or other studies that may affect your health, welfare, or willingness to stay in this study. If you want the results of the study, let the study staff know.

WHAT DO I DO IF I HAVE QUESTIONS OR PROBLEMS?

For questions about this study or a research-related injury, contact:

If you have any questions about this research study or you think you have a study-related injury, please feel free to contact Dr. David Haas or his research staff at 467-0154 during business hours. Should you have a problem in the evening or over the weekend, you may page the on-call staff at 835-9809.

For questions about your rights as a research subject, contact:

If you would like to find out more about giving consent or your rights as a participant in this study, please feel free to contact the Vanderbilt University Institutional Review Board (IRB) Office at (615) 322-2918 or toll free at (866) 224-8273. (An IRB is a group of people that reviews research studies to ensure participants’ rights and safety are protected.)

SIGNATURE PAGE

If you have read this consent form (or had it explained to you), all your questions have been answered and you agree to take part in this study, please sign your name below.

Participant’s Name (Print) Participant’s Signature and Date

Study Staff Conducting Study Staff Signature and Date

Consent Discussion (Print)

________________________ ______________________________

Witness’s Name (Print) Witness’s Signature and Date

(As appropriate)

# Consent for Genetic Research

The purpose of this study is to look at genes (DNA) and how they affect health and disease. Genes are the instruction manual for your body. The genes you get from your parents decide what you look like and how your body behaves. They can also tell us a person’s risk for certain diseases and how they will respond to treatment.

You are being asked to give a blood sample for genetic research. What we learn about you from this sample will not be put in your health record. Your test results will not be shared with you or your doctor. No one else (like a relative, boss, or insurance company) will be given your test results. Your sample will only be used for research at Vanderbilt University and will not be sold.

One risk of giving samples for this research may be the release of your name that could link you to the stored samples and/or the results of the tests run on your samples. This may cause problems with insurance or getting a job. To prevent this, these samples will be given a code. Only the study staff will know the code. The name that belongs to the code will be kept in a locked file or in a computer with a password. Only Dr. Haas and his research staff will have access to your name.

Your sample will be used to make DNA that will be kept for an unknown length of time (maybe years) for future research. The sample will be destroyed when it is no longer needed.

Your samples will be used for research only and will not be sold or used to make products that could be sold for money.

Your samples and information about you may be shared with others to use for research. To protect your privacy, we will not release your name.

You will not receive any benefit as a result of the tests done on your samples. These tests may help us learn more about the causes, risks, treatments, or how to prevent AIDS and other health problems.

At any time, you may ask to have your sample destroyed. You should contact Dr. Haas or study staff at 467-0154 to have your sample destroyed and no longer used for research. We will not be able to destroy research data that has already been gathered using your sample. Also, if your identity was removed from the samples, we will not be able to locate and destroy them.

There will be no costs to you for any of the tests done on your samples.

Please check Yes or No to the following question below:

My blood/tissue sample may be stored/shared for future gene research for other health problems (such as cancer, heart disease, etc).

Yes  No

SIGNATURE PAGE

If you have read this consent form (or had it explained to you), all your questions have been answered and you agree to take part in this study, please sign your name below.

Participant’s Name (Print) Participant’s Signature and Date

Study Staff Conducting Study Staff Signature and Date

Consent Discussion (Print)

________________________ ______________________________

Witness’s Name (Print) Witness’s Signature and Date

(As appropriate)
