# Supplementary material for: Genetic and Non-Genetic Determinants of Raltegravir Penetration into Cerebrospinal Fluid: A Single Arm Pharmacokinetic Study
Source: PLoS One. 2013 Dec 11;8(12):e82672. doi: 10.1371/journal.pone.0082672 (PMC3859605; doi:10.1371/journal.pone.0082672)
Supplement: Table S1 — Listing of all polymorphisms assayed, minor allele frequencies, and beta and P-values for association with CSF-to-plasma AUC0-4h ratios in study participants. (DOC) [file pone.0082672.s003.doc]

Supplemental On-line Table S1. Polymorphisms used in genotyping, minor allele frequencies in study subjects, and associations with CSF/AUC_0-4h_.

| SNP | CHR | Gene | MAF | Participants^a^ | Corrected Permutation Derived P^c^ | P^d^ | Beta^d^  (95% CI) |
| --- | --- | --- | --- | --- | --- | --- | --- |
| rs9869029 | 3 | *ABCC5* | 0.4875 | 40 | 1 | 0.154 | 0.245  (-0.084, 0.574) |
| rs3749442 | 3 | *ABCC5* | 0.1625 | 40 | 1 | 0.1959 | 0.219  (-0.106, 0.544) |
| rs3805114 | 3 | *ABCC5* | 0.1 | 40 | 1 | 0.287 | 0.182  (-0.148, 0.512) |
| rs1132776 | 3 | *ABCC5* | 0.4875 | 40 | 1 | 0.3039 | 0.181  (-0.159, 0.52) |
| rs56373140 | 3 | *ABCC5* | 0.0875 | 40 | 1 | 0.4086 | 0.135  (-0.181, 0.45) |
| rs3749445 | 3 | *ABCC5* | 0.375 | 40 | 1 | 0.4934 | 0.118  (-0.216, 0.452) |
| rs11718456 | 3 | *ABCC5* | 0.0125 | 40 | 1 | 0.5066 | 0.11  (-0.212, 0.432) |
| rs3749440 | 3 | *ABCC5* | 0.3125 | 40 | 1 | 0.8562 | -0.03  (-0.357, 0.296) |
| rs74711855 | 3 | *ABCC5* | 0 | 40 | NA | NA | NA |
| rs75617395 | 3 | *ABCC5* | 0 | 40 | NA | NA | NA |
| rs80282281 | 3 | *ABCC5* | 0 | 40 | NA | NA | NA |
| rs562 ^e^ | 3 | *ABCC5* | 0.375 | 40 | NA | NA | NA |
| rs939336 ^e^ | 3 | *ABCC5* | 0.4875 | 40 | NA | NA | NA |
| rs1316301 | 3 | *SLC15A2* | 0.3625 | 40 | 1 | 0.1662 | -0.242  (-0.578, 0.093) |
| rs4285028 | 3 | *SLC15A2* | 0.2625 | 40 | 1 | 0.1769 | 0.231  (-0.097, 0.558) |
| rs1136995 | 3 | *SLC15A2* | 0.125 | 40 | 1 | 0.3651 | -0.151  (-0.474, 0.172) |
| rs1920310 | 3 | *SLC15A2* | 0.0875 | 40 | 1 | 0.7507 | 0.065  (-0.334, 0.465) |
| rs1920311 | 3 | *SLC15A2* | 0 | 40 | NA | NA | NA |
| rs1920312 | 3 | *SLC15A2* | 0 | 40 | NA | NA | NA |
| rs2332043 | 3 | *SLC15A2* | 0 | 40 | NA | NA | NA |
| rs2689275 | 3 | *SLC15A2* | 0 | 40 | NA | NA | NA |
| rs60598279 | 3 | *SLC15A2* | 0 | 40 | NA | NA | NA |
| rs77229920 | 3 | *SLC15A2* | 0 | 40 | NA | NA | NA |
| rs77368557 | 3 | *SLC15A2* | 0 | 40 | NA | NA | NA |
| rs77782290 | 3 | *SLC15A2* | 0 | 40 | NA | NA | NA |
| rs79052538 | 3 | *SLC15A2* | 0 | 40 | NA | NA | NA |
| rs2293616 ^e^ | 3 | *SLC15A2* | 0.36 | 40 | NA | NA | NA |
| rs1143670 ^e^ | 3 | *SLC15A2* | 0.3625 | 40 | NA | NA | NA |
| rs1143671 ^e^ | 3 | *SLC15A2* | 0.3625 | 40 | NA | NA | NA |
| rs2622604 | 4 | *ABCG2* | 0.325 | 40 | 1 | 0.09892 | 0.266  (-0.041, 0.572) |
| rs2231137 | 4 | *ABCG2* | 0.0375 | 40 | 1 | 0.2758 | -0.194  (-0.536, 0.149) |
| rs17731538 | 4 | *ABCG2* | 0.125 | 40 | 1 | 0.2917 | -0.174  (-0.492, 0.144) |
| rs2231142 | 4 | *ABCG2* | 0.1125 | 40 | 1 | 0.3266 | -0.164  (-0.486, 0.159) |
| rs13120400 | 4 | *ABCG2* | 0.3875 | 40 | 1 | 0.3322 | 0.166  (-0.165, 0.497) |
| rs10030206 | 4 | *ABCG2* | 0 | 40 | NA | NA | NA |
| rs72552713 | 4 | *ABCG2* | 0 | 40 | NA | NA | NA |
| rs74362438 | 4 | *ABCG2* | 0 | 40 | NA | NA | NA |
| rs316003 | 6 | *SLC22A2* | 0.2125 | 40 | 1 | 0.1992 | -0.213  (-0.532, 0.106) |
| rs2450975 | 6 | *SLC22A2* | 0.2 | 40 | 1 | 0.2423 | -0.202  (-0.533, 0.13) |
| rs316019 | 6 | *SLC22A2* | 0.1 | 40 | 1 | 0.2799 | -0.181  (-0.505, 0.142) |
| rs694812 | 6 | *SLC22A2* | 0.0875 | 40 | 1 | 0.4307 | -0.139  (-0.48, 0.202) |
| rs3127594 | 6 | *SLC22A2* | 0.1125 | 40 | 1 | 0.4464 | -0.127  (-0.449, 0.196) |
| rs3127573 | 6 | *SLC22A2* | 0.125 | 40 | 1 | 0.4944 | -0.117  (-0.448, 0.214) |
| rs624249 | 6 | *SLC22A2* | 0.4375 | 40 | 1 | 0.9761 | -0.005  (-0.329, 0.319) |
| rs9355797 | 6 | *SLC22A2* | 0 | 40 | NA | NA | NA |
| rs3103353^e^ | 6 | *SLC22A2* | 0.1125 | 40 | NA | NA | NA |
| rs3127592^e^ | 6 | *SLC22A2* | 0.1125 | 40 | NA | NA | NA |
| rs2076828 | 6 | *SLC22A3* | 0.3625 | 40 | 1 | 0.1595 | 0.226  (-0.082, 0.533) |
| rs9457880 | 6 | *SLC22A3* | 0.075 | 40 | 1 | 0.2217 | -0.196  (-0.504, 0.112) |
| rs1810126 | 6 | *SLC22A3* | 0.375 | 40 | 1 | 0.8289 | -0.036  (-0.359, 0.287) |
| rs62440430 | 6 | *SLC22A3* | 0.075 | 40 | 1 | 0.8325 | -0.035  (-0.354, 0.285) |
| rs1050908 | 6 | *SLC22A3* | 0 | 40 | NA | NA | NA |
| rs74456818 | 6 | *SLC22A3* | 0 | 40 | NA | NA | NA |
| rs75498390 | 6 | *SLC22A3* | 0 | 40 | NA | NA | NA |
| rs77821354 | 6 | *SLC22A3* | 0 | 40 | NA | NA | NA |
| rs78360742 | 6 | *SLC22A3* | 0 | 40 | NA | NA | NA |
| rs80154852 | 6 | *SLC22A3* | 0 | 40 | NA | NA | NA |
| rs62568989 ^e^ | 6 | *SLC22A3* | 0.125 | 40 | NA | NA | NA |
| rs2048327 ^e^ | 6 | *SLC22A3* | 0.375 | 40 | NA | NA | NA |
| rs2292334 ^e^ | 6 | *SLC22A3* | 0.375 | 40 | NA | NA | NA |
| rs4437575 | 7 | *ABCB1* | 0.4697 | 132 | 1 | 0.2234 | -0.199  (-0.513, 0.115) |
| rs1045642 | 7 | *ABCB1* | 0.5 | 40 | 1 | 0.273 | -0.181  (-0.499, 0.137) |
| rs1055302 | 7 | *ABCB1* | 0.125 | 132 | 1 | 0.2746 | 0.174  (-0.133, 0.482) |
| rs1978095 | 7 | *ABCB1* | 0.007576 | 132 | 1 | 0.323 | -0.164  (-0.486, 0.157) |
| rs17209837 | 7 | *ABCB1* | 0.1477 | 132 | 1 | 0.3415 | 0.154  (-0.159, 0.468) |
| rs6978925 | 7 | *ABCB1* | 0.2576 | 132 | 1 | 0.3505 | -0.15  (-0.46, 0.161) |
| rs4148743 | 7 | *ABCB1* | 0.4508 | 132 | 1 | 0.3885 | 0.144  (-0.179, 0.466) |
| rs10248420 | 7 | *ABCB1* | 0.2875 | 40 | 1 | 0.3933 | -0.146  (-0.476, 0.185) |
| rs7787082 | 7 | *ABCB1* | 0.2875 | 40 | 1 | 0.3933 | -0.146  (-0.476, 0.185) |
| rs1016793 | 7 | *ABCB1* | 0.3636 | 132 | 1 | 0.3933 | 0.14  (-0.177, 0.458) |
| rs6969155 | 7 | *ABCB1* | 0.3902 | 132 | 1 | 0.417 | 0.137  (-0.19, 0.463) |
| rs4148738 | 7 | *ABCB1* | 0.4084 | 131 | 1 | 0.4311 | 0.128  (-0.186, 0.441) |
| rs868755 | 7 | *ABCB1* | 0.3712 | 132 | 1 | 0.4403 | 0.125  (-0.189, 0.44) |
| rs10276603 | 7 | *ABCB1* | 0.1477 | 132 | 1 | 0.4676 | -0.12  (-0.439, 0.2) |
| rs3789244 | 7 | *ABCB1* | 0.3902 | 132 | 1 | 0.4693 | 0.12  (-0.202, 0.442) |
| rs10225464 | 7 | *ABCB1* | 0.3931 | 131 | 1 | 0.4693 | 0.12  (-0.202, 0.442) |
| rs10808071 | 7 | *ABCB1* | 0.1929 | 127 | 1 | 0.4786 | -0.118  (-0.441, 0.205) |
| rs6948766 | 7 | *ABCB1* | 0.4583 | 132 | 1 | 0.4808 | 0.117  (-0.205, 0.438) |
| rs2235015 | 7 | *ABCB1* | 0.2375 | 40 | 1 | 0.4838 | -0.115  (-0.434, 0.204) |
| rs17327442 | 7 | *ABCB1* | 0.1402 | 132 | 1 | 0.5143 | -0.108  (-0.427, 0.212) |
| rs12535512 | 7 | *ABCB1* | 0.3826 | 132 | 1 | 0.5204 | 0.107  (-0.216, 0.43) |
| rs3842 | 7 | *ABCB1* | 0.1477 | 132 | 1 | 0.5615 | 0.099  (-0.232, 0.429) |
| rs1858923 | 7 | *ABCB1* | 0.4318 | 132 | 1 | 0.563 | 0.097  (-0.229, 0.424) |
| rs3789243 | 7 | *ABCB1* | 0.475 | 40 | 1 | 0.5806 | 0.091  (-0.229, 0.412) |
| rs17064 | 7 | *ABCB1* | 0.025 | 40 | 1 | 0.5934 | -0.09  (-0.417, 0.237) |
| rs13233308 | 7 | *ABCB1* | 0.4192 | 130 | 1 | 0.6951 | 0.066  (-0.263, 0.395) |
| rs10267099 | 7 | *ABCB1* | 0.2803 | 132 | 1 | 0.7639 | 0.053  (-0.289, 0.395) |
| rs1922242 | 7 | *ABCB1* | 0.4394 | 132 | 1 | 0.7758 | -0.047  (-0.369, 0.275) |
| rs10236274 | 7 | *ABCB1* | 0.06439 | 132 | 1 | 0.8155 | -0.041  (-0.378, 0.297) |
| rs1922243 | 7 | *ABCB1* | 0.06439 | 132 | 1 | 0.8155 | -0.041  (-0.378, 0.297) |
| rs1002205 | 7 | *ABCB1* | 0.072 | 132 | 1 | 0.8374 | 0.037  (-0.311, 0.384) |
| rs2188525 | 7 | *ABCB1* | 0.0375 | 40 | 1 | 0.8376 | -0.035  (-0.363, 0.293) |
| rs28381820 | 7 | *ABCB1* | 0.3712 | 132 | 1 | 0.8898 | 0.024  (-0.313, 0.361) |
| rs1202186 | 7 | *ABCB1* | 0.3674 | 132 | 1 | 0.8927 | -0.024  (-0.366, 0.319) |
| rs1989831 | 7 | *ABCB1* | 0.3712 | 132 | 1 | 0.8927 | -0.024  (-0.366, 0.319) |
| rs6972098 | 7 | *ABCB1* | 0.07576 | 132 | 1 | 0.8965 | -0.021  (-0.342, 0.299) |
| rs2188531 | 7 | *ABCB1* | 0.07955 | 132 | 1 | 0.8965 | -0.021  (-0.342, 0.299) |
| rs28381868 | 7 | *ABCB1* | 0.04545 | 132 | 1 | 0.9016 | 0.021  (-0.305, 0.347) |
| rs2235023 | 7 | *ABCB1* | 0.05725 | 131 | 1 | 0.9016 | 0.021  (-0.305, 0.347) |
| rs10264990 | 7 | *ABCB1* | 0.3598 | 132 | 1 | 0.9357 | -0.014  (-0.342, 0.315) |
| rs28381850 | 7 | *ABCB1* | 0.04924 | 132 | 1 | 0.9609 | -0.009  (-0.354, 0.337) |
| rs955000 | 7 | *ABCB1* | 0.06439 | 132 | 1 | 0.9609 | -0.009  (-0.354, 0.337) |
| rs2235035 | 7 | *ABCB1* | 0.3485 | 132 | 1 | 0.961 | -0.008  (-0.336, 0.32) |
| rs2235047 | 7 | *ABCB1* | 0.04167 | 132 | 1 | 0.9703 | 0.006  (-0.319, 0.331) |
| rs17149792 | 7 | *ABCB1* | 0.0303 | 132 | 1 | 0.9875 | -0.003  (-0.326, 0.321) |
| rs956825 | 7 | *ABCB1* | 0.3447 | 132 | 1 | 0.9927 | 0.002  (-0.328, 0.331) |
| rs2032582 | 7 | *ABCB1* | triallelic | 40 | NA | NA | NA |
| rs4148740 | 7 | *ABCB1* | NA^b^ | 0 | NA | NA | NA |
| rs2235052 | 7 | *ABCB1* | 0 | 40 | NA | NA | NA |
| rs28364278 | 7 | *ABCB1* | 0 | 40 | NA | NA | NA |
| rs28381940 | 7 | *ABCB1* | 0 | 132 | NA | NA | NA |
| rs28401792 | 7 | *ABCB1* | 0 | 132 | NA | NA | NA |
| rs28401801 | 7 | *ABCB1* | 0 | 132 | NA | NA | NA |
| rs28401814 | 7 | *ABCB1* | 0 | 132 | NA | NA | NA |
| rs28401815 | 7 | *ABCB1* | 0 | 132 | NA | NA | NA |
| rs28401816 | 7 | *ABCB1* | 0 | 132 | NA | NA | NA |
| rs28401819 | 7 | *ABCB1* | 0 | 40 | NA | NA | NA |
| rs3747802 | 7 | *ABCB1* | 0 | 40 | NA | NA | NA |
| rs72552784 | 7 | *ABCB1* | 0 | 40 | NA | NA | NA |
| rs80153317 | 7 | *ABCB1* | 0 | 40 | NA | NA | NA |
| rs2032588 ^e^ | 7 | *ABCB1* | 0.04545 | 132 | NA | NA | NA |
| rs28381863^e^ | 7 | *ABCB1* | 0.04924 | 132 | NA | NA | NA |
| rs28381869^e^ | 7 | *ABCB1* | 0.06061 | 132 | NA | NA | NA |
| rs28381873^e^ | 7 | *ABCB1* | 0.06061 | 132 | NA | NA | NA |
| rs17149699^e^ | 7 | *ABCB1* | 0.06439 | 132 | NA | NA | NA |
| rs28381857^e^ | 7 | *ABCB1* | 0.06439 | 132 | NA | NA | NA |
| rs17149824^e^ | 7 | *ABCB1* | 0.07955 | 132 | NA | NA | NA |
| rs2157926^e^ | 7 | *ABCB1* | 0.07955 | 132 | NA | NA | NA |
| rs10280101 ^e^ | 7 | *ABCB1* | 0.2 | 40 | NA | NA | NA |
| rs11983225 ^e^ | 7 | *ABCB1* | 0.2 | 40 | NA | NA | NA |
| rs12720067 ^e^ | 7 | *ABCB1* | 0.2 | 40 | NA | NA | NA |
| rs28401781 ^e^ | 7 | *ABCB1* | 0.2 | 40 | NA | NA | NA |
| rs4148739 ^e^ | 7 | *ABCB1* | 0.2 | 40 | NA | NA | NA |
| rs2373589 ^e^ | 7 | *ABCB1* | 0.2083 | 132 | NA | NA | NA |
| rs28381958 ^e^ | 7 | *ABCB1* | 0.2121 | 132 | NA | NA | NA |
| rs1202172 ^e^ | 7 | *ABCB1* | 0.3674 | 132 | NA | NA | NA |
| rs1202174 ^e^ | 7 | *ABCB1* | 0.3674 | 132 | NA | NA | NA |
| rs1202179 ^e^ | 7 | *ABCB1* | 0.3674 | 132 | NA | NA | NA |
| rs6959435 ^e^ | 7 | *ABCB1* | 0.3826 | 132 | NA | NA | NA |
| rs10276036 ^e^ | 7 | *ABCB1* | 0.3875 | 40 | NA | NA | NA |
| rs1128503 ^e^ | 7 | *ABCB1* | 0.3875 | 40 | NA | NA | NA |
| rs2235020 ^e^ | 7 | *ABCB1* | 0.3902 | 132 | NA | NA | NA |
| rs4148736 ^e^ | 7 | *ABCB1* | 0.4394 | 132 | NA | NA | NA |
| rs6961882 ^e^ | 7 | *ABCB1* | 0.4394 | 132 | NA | NA | NA |
| rs7802773 ^e^ | 7 | *ABCB1* | 0.4651 | 129 | NA | NA | NA |
| rs4149170 | 11 | *SLC22A6* | 0.1 | 40 | 1 | 0.6232 | 0.083  (-0.246, 0.412) |
| rs4149171 | 11 | *SLC22A6* | 0.1625 | 40 | 1 | 0.9027 | -0.02  (-0.342, 0.301) |
| rs2276299 | 11 | *SLC22A8* | 0.125 | 40 | 1 | 0.5119 | 0.109  (-0.214, 0.433) |
| rs4149180 | 11 | *SLC22A8* | 0.0125 | 40 | 1 | 0.843 | 0.034  (-0.297, 0.365) |
| rs4149179 | 11 | *SLC22A8* | 0 | 40 | NA | NA | NA |
| rs74582099 | 11 | *SLC22A8* | 0 | 40 | NA | NA | NA |
| rs12422149 | 11 | *SLCO2B1* | 0.125 | 40 | 1 | 0.2351 | -0.192  (-0.502, 0.119) |
| rs2712808 | 11 | *SLCO2B1* | 0.3625 | 40 | 1 | 0.3479 | -0.154  (-0.47, 0.163) |
| rs41298117 | 11 | *SLCO2B1* | 0.025 | 40 | 1 | 0.6587 | -0.072  (-0.39, 0.246) |
| rs2712810 | 11 | *SLCO2B1* | 0.15 | 40 | 1 | 0.6648 | -0.074  (-0.403, 0.256) |
| rs1801906 | 11 | *SLCO2B1* | 0.0375 | 40 | 1 | 0.6923 | -0.064  (-0.38, 0.251) |
| rs3781727 | 11 | *SLCO2B1* | 0.0125 | 40 | 1 | 0.9547 | -0.009  (-0.328, 0.31) |
| rs12278213 | 11 | *SLCO2B1* | NA^b^ | 0 | NA | NA | NA |
| rs79278361 | 11 | *SLCO2B1* | 0 | 40 | NA | NA | NA |
| rs79468961 | 11 | *SLCO2B1* | 0 | 40 | NA | NA | NA |
| rs2306168^e^ | 11 | *SLCO2B1* | 0.0375 | 40 | NA | NA | NA |
| rs10841781 | 12 | *SLCO1A2* | 0.125 | 40 | 1 | 0.1864 | -0.225  (-0.551, 0.102) |
| rs11045919 | 12 | *SLCO1A2* | 0.1625 | 40 | 1 | 0.1917 | 0.223  (-0.105, 0.551) |
| rs28446944 | 12 | *SLCO1A2* | 0.0625 | 40 | 1 | 0.2184 | -0.222  (-0.569, 0.125) |
| rs4149006 | 12 | *SLCO1A2* | 0.1125 | 40 | 1 | 0.249 | -0.192  (-0.511, 0.129) |
| rs4140389 | 12 | *SLCO1A2* | 0.175 | 40 | 1 | 0.2617 | 0.19  (-0.136, 0.516) |
| rs875234 | 12 | *SLCO1A2* | 0.175 | 40 | 1 | 0.2617 | 0.19  (-0.136, 0.516) |
| rs5488 | 12 | *SLCO1A2* | 0.2 | 40 | 1 | 0.5126 | -0.107  (-0.426, 0.211) |
| rs10841803 | 12 | *SLCO1A2* | 0.1 | 40 | 1 | 0.6831 | 0.069  (-0.259, 0.396) |
| rs3834939 | 12 | *SLCO1A2* | 0.3 | 40 | 1 | 0.8315 | 0.037  (-0.301, 0.375) |
| rs4149009 | 12 | *SLCO1A2* | 0.2875 | 40 | 1 | 0.8632 | 0.03  (-0.304, 0.363) |
| rs78801100 | 12 | *SLCO1A2* | NA^b^ | 0 | NA | NA | NA |
| rs10841777 | 12 | *SLCO1A2* | 0 | 40 | NA | NA | NA |
| rs10841778 | 12 | *SLCO1A2* | 0 | 40 | NA | NA | NA |
| rs10841779 | 12 | *SLCO1A2* | 0 | 40 | NA | NA | NA |
| rs10841780 | 12 | *SLCO1A2* | 0 | 40 | NA | NA | NA |
| rs12230113 | 12 | *SLCO1A2* | 0 | 40 | NA | NA | NA |
| rs12581327 | 12 | *SLCO1A2* | 0 | 40 | NA | NA | NA |
| rs4149004 | 12 | *SLCO1A2* | 0 | 40 | NA | NA | NA |
| rs71446763 | 12 | *SLCO1A2* | 0 | 40 | NA | NA | NA |
| rs73250843 | 12 | *SLCO1A2* | 0 | 40 | NA | NA | NA |
| rs74066041 | 12 | *SLCO1A2* | 0 | 40 | NA | NA | NA |
| rs77441275 | 12 | *SLCO1A2* | 0 | 40 | NA | NA | NA |
| rs78588104 | 12 | *SLCO1A2* | 0 | 40 | NA | NA | NA |
| rs11045916 ^e^ | 12 | *SLCO1A2* | 0.1125 | 40 | NA | NA | NA |
| rs11045917 ^e^ | 12 | *SLCO1A2* | 0.1125 | 40 | NA | NA | NA |
| rs12317843 ^e^ | 12 | *SLCO1A2* | 0.1625 | 40 | NA | NA | NA |
| rs61926244 ^e^ | 12 | *SLCO1A2* | 0.1625 | 40 | NA | NA | NA |
| rs1056007 ^e^ | 12 | *SLCO1A2* | 0.2 | 40 | NA | NA | NA |
| rs2291075 | 12 | *SLCO1B1* | 0.4375 | 40 | 1 | 0.3142 | 0.168  (-0.154, 0.49) |
| rs4149056 | 12 | *SLCO1B1* | 0.1375 | 40 | 1 | 0.4264 | 0.132  (-0.189, 0.454) |
| rs2306283 | 12 | *SLCO1B1* | 0.4375 | 40 | 1 | 0.5662 | 0.097  (-0.232, 0.426) |
| rs4149088 | 12 | *SLCO1B1* | 0.3625 | 40 | 1 | 0.6614 | 0.072  (-0.248, 0.392) |
| rs11045819 | 12 | *SLCO1B1* | 0.2 | 40 | 1 | 0.9776 | -0.005  (-0.329, 0.32) |
| rs4149085 | 12 | *SLCO1B1* | 0 | 40 | NA | NA | NA |
| rs4149087 ^e^ | 12 | *SLCO1B1* | 0.3625 | 40 | NA | NA | NA |
| rs16923154 | 12 | *SLCO1C1* | 0.15 | 40 | 1 | 0.1878 | -0.219  (-0.537, 0.1) |
| rs35209399 | 12 | *SLCO1C1* | 0.1625 | 40 | 1 | 0.2499 | -0.185  (-0.496, 0.125) |
| rs71581956 | 12 | *SLCO1C1* | 0.0625 | 40 | 1 | 0.2543 | -0.184  (-0.494, 0.127) |
| rs34243130 | 12 | *SLCO1C1* | 0.0625 | 40 | 1 | 0.4039 | -0.14  (-0.465, 0.185) |
| rs10841611 | 12 | *SLCO1C1* | 0.425 | 40 | 1 | 0.4677 | 0.119  (-0.199, 0.438) |
| rs10841613 | 12 | *SLCO1C1* | 0.4125 | 40 | 1 | 0.5368 | 0.101  (-0.217, 0.42) |
| rs7295794 | 12 | *SLCO1C1* | 0.425 | 40 | 1 | 0.8323 | 0.035  (-0.287, 0.357) |
| rs953002 | 12 | *SLCO1C1* | NA^b^ | 0 | NA | NA | NA |
| rs10444412 ^e^ | 12 | *SLCO1C1* | 0.4125 | 40 | NA | NA | NA |
| rs6487138 ^e^ | 12 | *SLCO1C1* | 0.4125 | 40 | NA | NA | NA |
| rs57270423 | 13 | *ABCC4* | 0.2125 | 40 | 0.982 | 0.0461 | -0.332  (-0.645, -0.018) |
| rs112792420 | 13 | *ABCC4* | 0.1125 | 40 | 1 | 0.1235 | 0.256  (-0.062, 0.574) |
| rs1557070 | 13 | *ABCC4* | 0.0125 | 40 | 1 | 0.2703 | 0.189  (-0.141, 0.519) |
| rs2274407 | 13 | *ABCC4* | 0.0625 | 40 | 1 | 0.3042 | -0.165  (-0.476, 0.145) |
| rs11568658 | 13 | *ABCC4* | 0.0125 | 40 | 1 | 0.323 | -0.164  (-0.486, 0.157) |
| rs6650282 | 13 | *ABCC4* | 0.45 | 40 | 1 | 0.3359 | -0.159  (-0.479, 0.16) |
| rs4148551 | 13 | *ABCC4* | 0.375 | 40 | 1 | 0.3629 | 0.157  (-0.177, 0.491) |
| rs3742106 | 13 | *ABCC4* | 0.3625 | 40 | 1 | 0.3812 | 0.149  (-0.181, 0.479) |
| rs2274406 | 13 | *ABCC4* | 0.3375 | 40 | 1 | 0.3855 | 0.145  (-0.178, 0.468) |
| rs3770 | 13 | *ABCC4* | 0.3875 | 40 | 1 | 0.4834 | 0.121  (-0.213, 0.454) |
| rs2274405 | 13 | *ABCC4* | 0.325 | 40 | 1 | 0.5134 | 0.108  (-0.212, 0.428) |
| rs3765534 | 13 | *ABCC4* | 0.025 | 40 | 1 | 0.5838 | 0.092  (-0.234, 0.417) |
| rs1678339 | 13 | *ABCC4* | 0.0125 | 40 | 1 | 0.6262 | -0.081  (-0.405, 0.243) |
| rs899494 | 13 | *ABCC4* | 0.1 | 40 | 1 | 0.7606 | 0.05  (-0.269, 0.369) |
| rs1059751 | 13 | *ABCC4* | 0.4375 | 40 | 1 | 0.841 | 0.035  (-0.305, 0.375) |
| rs9561778 | 13 | *ABCC4* | 0.2125 | 40 | 1 | 0.9582 | 0.009  (-0.321, 0.339) |
| rs11568655 | 13 | *ABCC4* | 0.0125 | 40 | 1 | 0.9586 | 0.009  (-0.318, 0.336) |
| rs1926657 | 13 | *ABCC4* | 0.1125 | 40 | 1 | 0.9821 | -0.004  (-0.322, 0.314) |
| rs79725195 | 13 | *ABCC4* | 0 | 40 | NA | NA | NA |
| rs11568695 | 13 | *ABCC4* | 0.0125 | 40 | NA | NA | NA |
| rs1189466 | 13 | *ABCC4* | 0.0125 | 40 | NA | NA | NA |
| rs1729747 ^e^ | 13 | *ABCC4* | 0.2125 | 40 | NA | NA | NA |
| rs1289389 | 13 | *SLC15A1* | 0.2 | 40 | 0.6723 | 0.007345 | 0.446  (0.14, 0.753) |
| rs1339067 | 13 | *SLC15A1* | 0.2875 | 40 | 0.983 | 0.04677 | 0.334  (0.017, 0.652) |
| rs4646234 | 13 | *SLC15A1* | 0.1 | 40 | 1 | 0.4574 | 0.127  (-0.205, 0.459) |
| rs8187832 | 13 | *SLC15A1* | 0.025 | 40 | 1 | 0.7032 | -0.062  (-0.378, 0.254) |
| rs2297322 | 13 | *SLC15A1* | 0.075 | 40 | 1 | 0.785 | 0.047  (-0.286, 0.379) |
| rs74900212 | 13 | *SLC15A1* | 0 | 40 | NA | NA | NA |
| rs78068268 | 13 | *SLC15A1* | 0 | 40 | NA | NA | NA |
| rs78350626 | 13 | *SLC15A1* | 0 | 40 | NA | NA | NA |
| rs1517618 | 15 | *SLCO3A1* | 0.2375 | 40 | 0.9231 | 0.02525 | 0.383  (0.062, 0.704) |
| rs7496880 | 15 | *SLCO3A1* | 0.325 | 40 | 0.981 | 0.04529 | 0.343  (0.02, 0.666) |
| rs6496899 | 15 | *SLCO3A1* | 0.2179 | 39 | 0.99 | 0.05029 | 0.323  (0.011, 0.635) |
| rs9302356 | 15 | *SLCO3A1* | 0.3125 | 40 | 0.992 | 0.0531 | 0.325  (0.007, 0.644) |
| rs3743370 | 15 | *SLCO3A1* | 0.3375 | 40 | 1 | 0.08515 | 0.299  (-0.032, 0.63) |
| rs2270061 | 15 | *SLCO3A1* | 0.3125 | 40 | 1 | 0.1047 | -0.266  (-0.578, 0.047) |
| rs3030616 | 15 | *SLCO3A1* | 0.35 | 40 | 1 | 0.2239 | -0.198  (-0.51, 0.115) |
| rs3924426 | 15 | *SLCO3A1* | 0.025 | 40 | 1 | 0.2854 | -0.179  (-0.501, 0.144) |
| rs2286355 | 15 | *SLCO3A1* | 0.3375 | 40 | 1 | 0.3721 | -0.15  (-0.475, 0.175) |
| rs16946494 | 15 | *SLCO3A1* | 0.05 | 40 | 1 | 0.4678 | 0.123  (-0.206, 0.452) |
| rs1053909 | 15 | *SLCO3A1* | 0.4 | 40 | 1 | 0.5812 | -0.091  (-0.409, 0.228) |
| rs2108601 | 15 | *SLCO3A1* | 0.325 | 40 | 1 | 0.648 | -0.077  (-0.405, 0.251) |
| rs12594058 | 15 | *SLCO3A1* | 0 | 40 | NA | NA | NA |
| rs16946476 | 15 | *SLCO3A1* | 0 | 40 | NA | NA | NA |
| rs2302085 | 15 | *SLCO3A1* | 0 | 40 | NA | NA | NA |
| rs75482144 | 15 | *SLCO3A1* | 0 | 40 | NA | NA | NA |
| rs78641225 | 15 | *SLCO3A1* | 0 | 40 | NA | NA | NA |
| rs8030651 | 15 | *SLCO3A1* | 0 | 40 | NA | NA | NA |
| rs1060205^e^ | 15 | *SLCO3A1* | 0.05 | 40 | NA | NA | NA |
| rs11074043^e^ | 15 | *SLCO3A1* | 0.05 | 40 | NA | NA | NA |
| rs3743366^e^ | 15 | *SLCO3A1* | 0.05 | 40 | NA | NA | NA |
| rs59470206 ^e^ | 15 | *SLCO3A1* | 0.3125 | 40 | NA | NA | NA |
| rs8174 ^e^ | 15 | *SLCO3A1* | 0.3125 | 40 | NA | NA | NA |
| rs3784862 | 16 | *ABCC1* | 0.225 | 40 | 0.5495 | 0.004103 | 0.44  (0.16, 0.72) |
| rs35604 | 16 | *ABCC1* | 0.1625 | 40 | 0.999 | 0.07317 | 0.302  (-0.018, 0.622) |
| rs246240 | 16 | *ABCC1* | 0.1125 | 40 | 1 | 0.2128 | 0.201  (-0.109, 0.511) |
| rs4148380 | 16 | *ABCC1* | 0.0625 | 40 | 1 | 0.3981 | 0.148  (-0.191, 0.488) |
| rs3743527 | 16 | *ABCC1* | 0.2125 | 40 | 1 | 0.578 | -0.094  (-0.42, 0.233) |
| rs35592 | 16 | *ABCC1* | 0.175 | 40 | 1 | 0.7608 | -0.051  (-0.374, 0.273) |
| rs246221 | 16 | *ABCC1* | 0.2 | 40 | 1 | 0.8081 | 0.041  (-0.285, 0.366) |
| rs212090 | 16 | *ABCC1* | 0.425 | 40 | 1 | 0.8488 | 0.035  (-0.322, 0.392) |
| rs212091 | 16 | *ABCC1* | 0.15 | 40 | 1 | 0.8768 | 0.027  (-0.315, 0.37) |
| rs3765129 | 16 | *ABCC1* | 0.15 | 40 | 1 | 0.9006 | -0.02  (-0.338, 0.298) |
| rs35621 | 16 | *ABCC1* | 0.0625 | 40 | 1 | 0.97 | -0.006  (-0.327, 0.315) |
| rs45569938 | 16 | *ABCC1* | 0 | 40 | NA | NA | NA |
| rs80085493 | 16 | *ABCC1* | 0 | 40 | NA | NA | NA |
| rs8187856 | 16 | *ABCC1* | 0 | 40 | NA | NA | NA |
| 1. Number of participants successfully genotyped. Genotyping of *ABCB1* rs1045642 heterozygotes was only performed for selected SNPs within *ABCB1*. 2. Genotyping unsuccessful. 3. Empirically derived permutation adjusted P value. Value is corrected for BMI, age, CSF protein, and sex. 4. Linear regression derived Beta and P value, corrected for BMI, age, CSF protein, and sex. 5. NA indicates polymorphism in complete LD with another polymorphism in this dataset or not successfully genotyped, so therefore not analyzed separately. | | | | | | | |
